# The Noetic Signature Inventory: Development, Exploration, and Initial Validation

**DOI:** 10.3389/fpsyg.2022.838582

**Published:** 2022-06-15

**Authors:** Helané Wahbeh, Nina Fry, Paolo Speirn

**Affiliations:** Department of Research, Institute of Noetic Sciences, Petaluma, CA, United States

**Keywords:** intuition, noetic, validation, questionnaire, non-local consciousness, extended perception

## Abstract

Noetic comes from the Greek word noēsis, meaning inner wisdom or direct knowing. Noetic experiences often transcend the perception of our five senses and are ubiquitous worldwide, although no instrument exists to evaluate noetic characteristics both within and between individuals. We developed the Noetic Signature Inventory (NSI) through an iterative qualitative and statistical process as a tool to subjectively assess noetic characteristics. Study 1 developed and evaluated a 175-item NSI using 521 self-selected research participants, resulting in a 46-item NSI with an 11-factor model solution. Study 2 examined the 11-factor solution, construct validity, and test–retest reliability, resulting in a 44-item NSI with a 12-factor model solution. Study 3 confirmed the final 44-item NSI in a diverse population. The 12-factors were: (1) Inner Knowing, (2) Embodied Sensations, (3) Visualizing to Access or Affect, (4) Inner Knowing Through Touch, (5) Healing, (6) Knowing the Future, (7) Physical Sensations from Other People, (8) Knowing Yourself, (9) Knowing Other’s Minds, (10) Apparent Communication with Non-physical Beings, (11) Knowing Through Dreams, and (12) Inner Voice. The NSI demonstrated internal consistency, convergent and divergent content validity, and test–retest reliability. The NSI can be used for the future studies to evaluate intra- and inter-individual variation of noetic experiences.

## Introduction

Noetic comes from the Greek word noēsis/noētikos, meaning inner wisdom, direct knowing, intuition, or implicit understanding. Noetic refers to ways of knowing beyond our traditional five senses. Noetic information is perceived as different from information involving the intellect or information received through an individual’s five physical senses. In considering the noetic quality of mystical experiences, William James, the American philosopher and psychologist, described these experiences as “states of knowledge. They are states of insight into depths of truth unplumbed by the discursive intellect. They are illuminations, revelations, full of significance and importance, all inarticulate though they remain; and as a rule, they carry with them a curious sense of authority for after-time” (James, 1985, p. 380–381). Research into the noetic quality of religious, spiritual, and mystical experiences (Beauregard, 2011) describe the “perceptions of unity, ineffability, positive emotions, and sacredness” that often occur with such experiences (Yaden et al., 2017). Others have used the term noetic in a different context, such as Endel Tulving, who used the terms anoetic (non-knowing), noetic (knowing), and autonoetic (self-knowing) to delineate three kinds of conscious states, each associated with a distinct kind of memory (Tulving, 1985, 2002). For example, Tulving described that noetic consciousness “allows an organism to be aware of, and to cognitively operate on, objects and events, and relations among objects and events, in the absence of these objects and events” (Tulving, 1985, p. 4). In this paper, we use the term noetic as related by James, Beauregard, and Yaden, referring to the subjective experience of intuitively accessing knowledge beyond our physical senses and without intellectual analysis. We propose that all people access noetic information. However, the way in which each individual accesses this information is unique. Here, we detail the development and validation of the Noetic Signature Inventory (NSI), a measure of each individual’s unique combination of factors to their noetic experiences.

Noetic experiences appear ubiquitous and have been described globally despite their ineffable nature (McClenon, 1993; Haraldsson, 2011; Castro et al., 2014; Hunter and Luke, 2014; Wahbeh et al., 2018b). For example, between 34% and 80% of participants polled in multiple studies reported experiencing mind-to-mind communication across a distance (i.e., not using traditional means; Wahbeh et al., 2018b). Humanity’s oldest writings record such experiences in cultures worldwide (Hastings, 1991). In the fifth century BC, Socrates noted hearing an inner voice giving him a protective warning message. He was walking down a street in Athens with some friends and internally heard a message to go down a different street. He listened to the message, changed his walking path, and was safe. His friends did not heed his warning, and a herd of wild pigs came barreling down the road and knocked them down (Hastings, 1991, p. 119). These noetic experiences have had many terms ascribed to them, such as intuition, psychic, extended human capacities, anomalous information reception, telepathy, and clairaudience.

Some attribute these noetic phenomena to the unconscious mind, described as “the influences or effects of stimulus processing of which one is not aware” (Bargh and Morsella, 2008). Perhaps noetic experiences represent an unconscious perception of something greater than our personal selves that we are connected to that provides us with information to generate judgments, decisions, and reasoning unconsciously.

Sigmund Freud made the concept of the unconscious mind mainstream with his psychological theories and therapeutic methods. He likened our mind to an iceberg with only minimal mental activity within our conscious awareness. The subconscious below the water’s surface is not normally within our awareness, but we could potentially become aware of it. The unconscious was the bulk of the iceberg below the water’s surface and represented aspects of ourselves of which we were unaware and could not become aware. Freud thought the unconscious mind was the primary motivator of a person’s daily actions despite its veiled status. Freud’s model highlighted mostly sexual and aggressive impulses and fears as components of the unconscious (Bargh, 2019).

Others have supported more positive aspects of our unconscious. For example, Dr. Roberto Assagioli, a prominent psychologist, developed a model of the mind that includes the unconscious, which has lower, middle, and higher unconscious or superconscious aspects (Assagioli, 1965). Assagioli described the superconscious as encompassing noble qualities and highlighted its importance and recognition by a growing number of psychologists,

*Consequently some of the more advanced psychologists have recognized the existence, and have started the scientific study, of the superconscious, that is, of that psychospiritual realm where are organized and developed, and from whence penetrate into man’s consciousness, all higher inspirations, philosophical and scientific intuitions, spiritual illumination, telepathic impressions, exceptional healing powers and impulses to heroic and self-sacrificing deeds.* (Assagioli, 1965, p. 3).

The sequelae of tapping into the psychospiritual realm are the same noetic experiences we highlight here and deserve further study.

Beyond the personal unconscious, the collective unconscious is also relevant to noetic experiences. Jung proposed a collective unconscious that is the same and present in all humans, a connecting matrix of archetypes.

*In addition to our immediate consciousness, which is of a thoroughly personal nature and which we believe to be the only empirical psyche (even if we tack on the personal unconscious as an appendix), there exists a second psychic system of a collective, universal, and impersonal nature which is identical in all individuals. This collective unconscious does not develop individually but is inherited. It consists of pre-existent forms, the archetypes, which can only become conscious secondarily and which give definite form to certain psychic contents* (Jung, 1936, p. 99).

Jung’s collective unconscious extends beyond the personal self that all humans are intrinsically connected to, a model which can encompass noetic experiences. Interestingly, Jung experienced a dream, where a wise figure named Philemon appeared to him. Jung believed Philemon was an autonomous consciousness beyond his personal conscious or unconscious. He would regularly converse with and receive guidance and insight from him. This experience is reminiscent of what many people experience as spirit guides in other traditions and the noetic experience of communication with apparent non-physical beings. Later in Jung’s life, he connected with another inner guide name Ka. Jung believed these figures were products of the collective unconscious, beyond his personal self (Jung, 1975 as cited by Hastings, 1991, p. 115). Perhaps it is not the personal unconscious, but the collective unconscious that provides the basis for the noetic experiences regularly observed worldwide and in laboratories (Cardeña et al., 2015; Krippner, 2021).

Noetic experiences also show themselves in many contexts, from positive psychology to transpersonal psychology to spiritual emergence. Positive psychology focuses on the positive aspects of life and personality with a eudaimonic perspective, highlighting the sense of striving toward realizing one’s potential or goals, a life purpose, and seeking personal growth (Cannard et al., 2021). Noetic experiences inspire greater meaning in life. One study found that people who had noetic and/or transcendent experiences increased their interest in spirituality, sense of connection to others, happiness, well-being, confidence, optimism about the future, and meaning in life (Kennedy and Kanthamani, 1995a,b).

Transpersonal psychology also provides a more positive perspective on our conscious and unconscious personality, holding that it contains qualities such as intuition, creativity, purpose and meaning, higher values, transcendent experiences, and spiritual concern. Walsh and Vaughn suggest that these aspects of ourselves are part of our development as humans and that noetic experiences express our capacity to tap into these aspects of ourselves (Walsh and Vaughan, 1993). Transpersonal psychology also encompasses noetic experiences through its traditional focus on “transcendence: certain peak experiences or altered states of consciousness achieved spontaneously, or through spiritual practice, ritual, or mind-altering substance were seen as portals to beyond-ego levels or alternate metaphysical dimensions” (Hartelius et al., 2021, p. 10). Noetic experiences, by their nature, transcend the personal self. Also, altered states of consciousness are clear pathways to having noetic experiences, as people in altered states have more spontaneously and do better on noetic tasks in the laboratory (Bourguignon, 1973; Cardeña and Marcusson-Clavertz, 2015; Luke, 2022).

Many noetic experiences can also be viewed from the lens of spiritual emergence. Stanislav Grof, MD, and his wife Christina Grof coined the term spiritual emergence, representing the healing and transformative potential for crises on the spiritual path, resulting in a higher level of psychological functioning and spiritual awareness (Grof, 2019, p. 314). Spiritual traditions worldwide document these Dark Nights of the Soul, expect them, and offer guidance on navigating their often challenging and life-altering waters (Durà-Vilà and Dein, 2009). The Grof’s developed Holotropic Breathwork at the Esalen Institute in Big Sur, California, in the mid-1970s after studying the use of non-ordinary states of consciousness in various cultures and settings. Holotropic means: “holo” means wholeness, and “tropic” means moving toward; “moving toward wholeness” (Brewerton et al., 2012). Thus, practitioners are often initiated into spiritual emergence through the altered states of consciousness achieved through Holotropic Breathwork. The cross-section between noetic experiences and spiritual emergence lies in their similar content. For example, people in spiritual emergent states may connect with apparent non-physical beings, know information about others or the future that they would usually have no way of knowing through traditional means, or receive verifiable mental impressions from people or places at a distance (Corneille and Luke, 2021).

Despite their prevalence and potentially positive healing potential, academics, and clinicians across many disciplines are not willing to explore the possibility of noetic experiences in any tangible way. As Dr. Grof notes, many noetic experiences that “are currently diagnosed as psychoses and indiscriminately treated by suppressive medication are actually difficult states of a radical personality transformation and of spiritual opening. If they are correctly understood and supported, these psychospiritual crises can result in emotional and psychosomatic healing, remarkable transformation, and consciousness evolution. They also have great heuristic potential… Mainstream psychiatrists are unable to see the difference between psychospiritual crises, or even uncomplicated mystical states, and serious mental illness, because of their narrow conceptual framework” (Grof, 2019, p. 313–314). Unfortunately, academia has a similar negative view of noetic experiences. Scientific research into these topics is taboo in most Western academic settings (Cardeña, 2015; Schooler et al., 2018). Thus, it is unlikely that the volume of research on these topics is commensurate with their prevalence.

Despite this, growing objective evidence from laboratory studies demonstrates their observable and replicable nature (see Cardeña, 2018 for a review). For example, one noetic experience, remote viewing, is the ability to access mental impressions about distant people and places that one would not usually access. The United States military conducted a formal remote viewing program for more than two decades. Drs. May and Marwaha synthesized the data released about this program stating, “In a total of 504 separate missions from 1973 to 1995, remote viewing produced actionable intelligence prompting 89 percent of the customers to return with additional missions. The Star Gate data indicates that information psi is a scientifically valid phenomenon” (May and Marwaha, 2018a,b). This and other formal remote viewing studies result in significant meta-analytic effect sizes ranging from 0.17 to 0.39 (Cardeña, 2018). Remote viewing is just one noetic experience. Exploring the full breadth of the evidential research available on these phenomena is beyond the scope of this paper. Readers can consult the Handbook of Parapsychology (Cardeña et al., 2015) as a primer for the topic.

Despite any taboos or controversy about whether these experiences are “real” or not, these phenomena are commonly reported subjective experiences that are meaningful to the person having them (Wahbeh et al., 2018b). Recent psychological models suggest that noetic experiences are an innate human capacity: the Psi-Mediated Instrumental Response (PMIR; Stanford, 2015) and the First-Sight Model and Theory (FSMT; Carpenter, 2014). The PMIR model proposes that individuals may unconsciously obtain extrasensory information that they then used to modify their behavior adaptively. The FSMT proposes that humans’ essential nature is to actively, continuously, and unconsciously participate in the world, which extends beyond our immediate boundaries of perceived space and time. All our experiences and behaviors result from unconscious psychological processes that are acted out based on multiple sources of information, including those beyond our traditional five senses. According to both these models, some level of intuitive ability is likely available to all humans.

Additional research has examined the potential overlap between noetic experiences and paranormal beliefs and other constructs such as personality traits, empathy, sensory processing sensitivity, and mental health symptoms (e.g., psychosis and dissociation). Research has been mixed on the topic of personality and noetic experiences/paranormal beliefs (Williams et al., 2007; Luke et al., 2008; Hitchman et al., 2012; Miklousic et al., 2012; Chauvin and Mullet, 2021). Some studies have observed significant correlations between the noetic/paranormal and neuroticism, extraversion, conscientiousness, openness, withdrawal, industriousness, and assertiveness (Thalbourne and Haraldsson, 1980; Thalbourne, 1995; Thalbourne et al., 1995; Wiseman and Watt, 2004; Saucier and Skrzypińska, 2006; Swami et al., 2011; Cardeña et al., 2015), while others have not (Lester and Monaghan, 1995; Willging and Lester, 1997; Rattet and Bursik, 2001; Peltzer, 2002; Wahbeh et al., 2020b). Personality and noetic experiences are different constructs, even though some studies observe a correlation between the two. In addition, some researchers have hypothesized a positive association between paranormal experiences and sensory processing sensitivity (Greeley, 1975). The research on this relationship is also mixed and limited (Houran et al., 2002; Jawer, 2006), though some researchers have found a positive relationship between the two constructs (Kjellgren et al., 2009; Irwin et al., 2014; Carr, 2021). Additional studies have shown a positive relationship between paranormal experiences and empathy (McCreery and Claridge, 1995, 2002; Krippner et al., 2000; Irwin, 2017). Similarly, empathy and sensory processing sensitivity may be present in people with more noetic experiences but they too are not the same constructs.

Finally, research has indicated positive associations between paranormal beliefs/experiences and schizotypy (Windholz and Diamant, 1974; Wolfradt, 1997; Irwin and Green, 1999; Genovese, 2005; Hergovich et al., 2008; Dembihska-Krajewska and Rybakowski, 2014; Dagnall et al., 2016) as well as between paranormal beliefs/experiences and dissociation (Richards, 1991; Irwin, 1994; Zingrone and Alvarado, 1994; Sharps et al., 2006; Gow et al., 2009). Often the correlative relationships are driven by similarities in wording on schizotypy and dissociation self-report questionnaires. For example, one item on the Dissociative Experiences Scale—Taxon (Waller et al., 1996) is “Some people sometimes find that they hear voices inside their head which tell them to do things or comment on things that they are doing,” which someone might endorse who hears an apparent spiritual guide speaking to them and is otherwise highly functional and well-adjusted. Furthermore, it is important to note that although some psychotic and dissociative symptoms resemble noetic experiences (e.g., hearing voices or disembodied sensations), the crucial distinction is that the vast majority of people who have noetic experiences do not have pathological levels of these symptoms, are highly functional, and find the experiences meaningful and beneficial (Richards, 1991; Castillo, 2003; Moreira-Almeida et al., 2008; Seligman and Kirmayer, 2008; Moreira-Almeida and Koss-Chioino, 2009; Rabeyron and Watt, 2010; Moreira-Almeida and Cardeña, 2011; Roxburgh and Roe, 2011; Dein, 2012; Stolovy et al., 2015; Pederzoli et al., 2021).

In the current study, we hypothesize that each individual can tap into noetic information and has a unique way to experience noetic information. We call this an individual’s noetic signature. The specific way people experience the noetic is quite varied (Wahbeh et al., 2022). For example, one person may feel sensations in their body, what they might call a gut hunch when they receive intuitive information. Another person may get goosebumps on their skin, signaling them to pay attention to any perceived information. Another person may observe colors or energy movement around people. Still, others have dreams that offer them insight into life decisions. These examples represent characteristics that could make up someone’s noetic signature. Importantly, people can experience more than one type of characteristic, but some are usually stronger or more dominant than others. In order to more fully explore these noetic experiences, a valid and reliable instrument is needed.

Some instruments exist to evaluate whether people have had various noetic experiences or believe in these experiences, but each has its limitations (Gallagher et al., 1994; Kohls and Walach, 2006; Wahbeh et al., 2020b). The Anomalous Experiences Inventory (AEI) has 70 items comprised of five subscales: anomalous/paranormal experience, anomalous/paranormal belief, anomalous/paranormal ability, fear of the anomalous/paranormal, and use of drugs and alcohol. While the anomalous/paranormal experience and ability subscales of the AEI most clearly resemble the measure, we are proposing here a limitation of the AEI is the use of biased language for many of the included items. For example, one question is “I am psychic,” which is problematic since “psychic” may mean different things to different people. The Exceptional Experiences Questionnaire is a 57-item questionnaire with four subscales: positive spiritual experiences, loss of ego/deconstruction, psychopathology, and dreams (Kohls and Walach, 2006). This questionnaire evaluates the frequency and perceived impact of these experiences. The Noetic Experiences and Belief Scale (NEBS) measures the belief and experience of 10 paranormal phenomena: intuition, non-local consciousness, extraterrestrials, precognition, life after death, contact with the dead, clairvoyance, psychokinesis, telepathy, and automatism (Wahbeh et al., 2020b). While the NEBS addresses noetic beliefs and experiences, the 10 items are not focused on the subjective personal expression of accessing knowledge beyond our physical senses and without intellectual analysis. These measures essentially provide a measure for an individual’s belief in and experience with noetic information. However, none of these measures explores the nuanced way in which an individual receives and experiences noetic information to provide a personal *profile* of noetic characteristics.

This research project’s overall goal was to develop a self-report questionnaire, the NSI, to evaluate people’s noetic signatures with less than 50-items for ease of administration and test its validity and test–retest reliability. The NSI will measure an individual’s unique experience of noetic information, allowing for evaluations of the intra- and inter-individual variability of noetic characteristics. As the first step in developing and validating the NSI, we conducted a survey to gain a detailed report of qualitative first-person accounts of noetic characteristics (Wahbeh et al., 2022). Thematic analysis was conducted to characterize the data.

**Figure 1:**
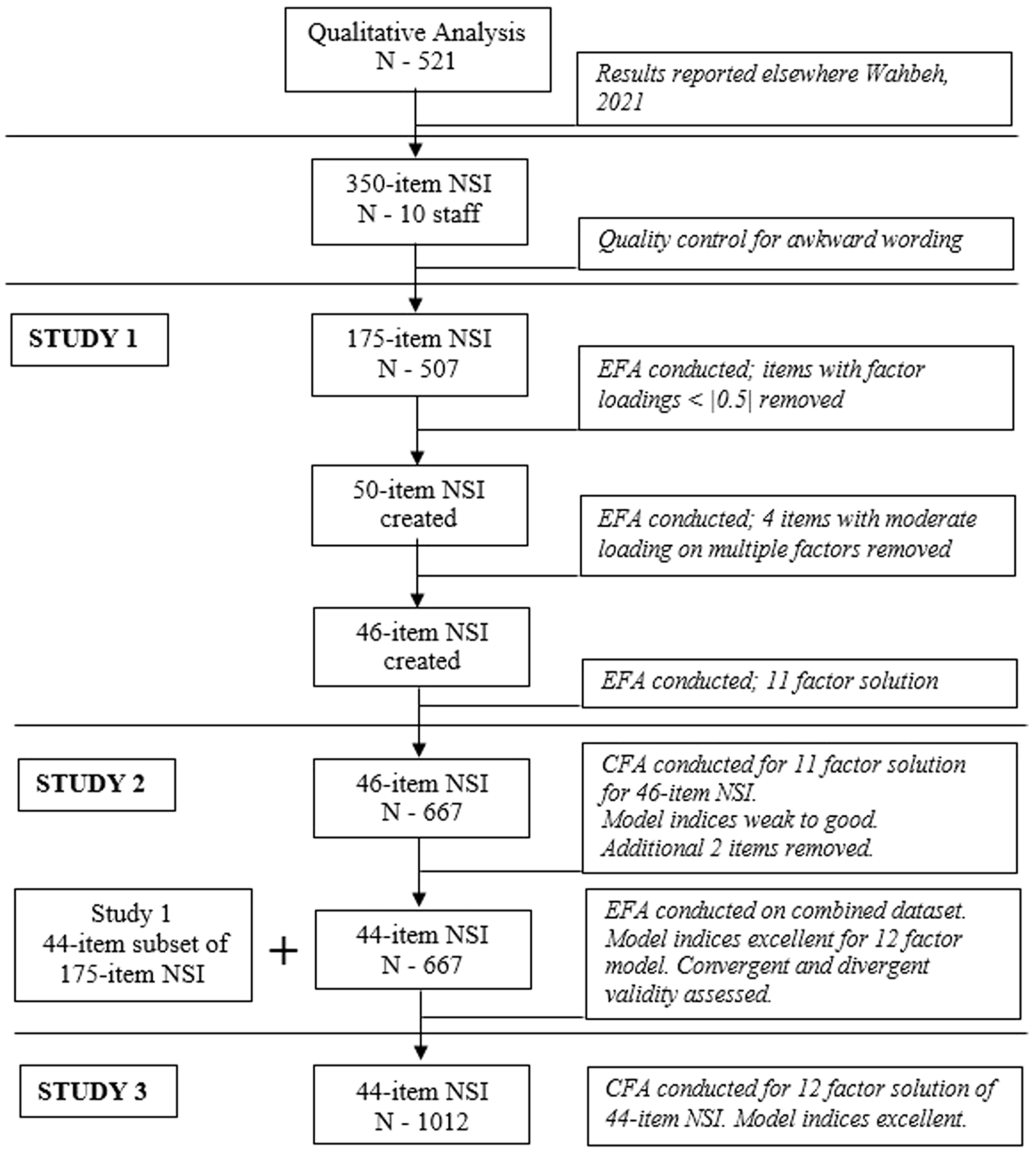
Summary of Studies. Schematic summarizing studies including participant number for each study, analyses conducted, and changes made to NSI at each step.

Building on the themes derived from this qualitative analysis, we conducted three additional studies to develop and validate the NSI (see Figure 1 for study flow). Study 1 developed and evaluated a 175-item version and resulted in a 46-item version NSI with an 11-factor exploratory factor analysis (EFA) solution. Study 2 attempted to confirm the 11-factor solution, evaluated construct validity, and explored test–retest reliability. Study 2 resulted in a 44-item NSI with a 12-factor EFA model solution. Study 3 then conducted a confirmatory factor analysis (CFA) of the final 44-item NSI in a separate and more diverse population. The Institute of Noetic Sciences (IONS) Institutional Review Board (approval designation WAHH_2019_01) approved all study activities.

## Study 1: Development of a 46-Item NSI

This study aimed to develop a preliminary version of the NSI based on the results of a qualitative analysis of first-person accounts of noetic experiences reported in Wahbeh et al. (2022).

### Methods

#### Participants

Nine hundred and eighty participants were recruited from IONS membership through e-newsletters, blogs, and an online recruitment posting. Inclusion criteria were: (1) age 18 years or older, (2) fluent in English, and (3) having had a prior noetic experience. Specific language for this inclusion criterion was as follows: “In this study, IONS wants to learn about people’s experiences with information or energy not limited by conventional notions of space and time. It is traditionally accepted that we can gain information from our traditional five senses—sight, smell, taste, touch, and hearing. We are asking about your experiences with information or energy from beyond these traditional five senses. Many people feel like some of these experiences are also outside of our traditional accepted understanding of time. Some examples of these types of experiences are: knowing another person’s thoughts without them sharing them with you, or just knowing something about someone that you could not possibly know otherwise. These are just two examples, there are many more. IONS wants to learn about YOUR unique experiences. Have you had any experiences with information or energy not limited by conventional notions of space and time?” Participants had to mark YES to this question to continue.

All participants signed an informed consent to participate in the study consistent with the IONS Institutional Review Board guidelines. Participants were entered into a $100 gift card raffle for their participation. Demographic information was inadvertently not collected from participants included in this study.

#### Measures and Procedures

As the first step to developing the NSI, we conducted a qualitative study with 521 English-speaking adults worldwide. The detailed results of this preliminary study are reported elsewhere (Wahbeh et al., 2022). Participants who self-identified as having noetic experiences completed an online survey that collected demographic data and four open-ended questions about noetic experiences. Thematic analysis was conducted to characterize the data. The goal was to gain insight into people’s unique expressions of their noetic experiences. Five main themes were identified: (1) Ways of Engagement, (2) Ways of Knowing, (3) Types of Information, (4) Ways of Affecting, and (5) Ways of Expressing. Ten most used subthemes were expressing to or sharing with others, impacting decision-making, intuition/“just knowing,” meditation/hypnosis, inner visions, setting intentions/getting into the “state,” healing others, writing for self, and inner voice. Using Dedoose web-based qualitative data analysis software (version 8.3.17, Dedoose, Inc., Hermosa Beach, CA), 78 codes were generated from this qualitative data set as reported in Wahbeh et al. (2022).

From these results, we created five questions decided by author consensus for each identified code using the following guidelines: use simple language, create specific questions that can apply to a wide range of respondents, avoid double negatives, avoid double-barreled questions, and avoid absolutes. All items were also worded in the past tense to capture lifetime experiences. The tense of the items and answer choices reflected our aim to develop an inventory that assessed characteristics rather than frequency of experiences. Items included such statements as, “I have received communication directly from other people’s minds,” and “I have received information about things that will happen in the future.” We used a sliding scale from 0 to 100 anchored by Strongly Disagree (0), Neither Agree Nor Disagree (50), and Strongly Agree (100) as each item’s answer choice. This process resulted in a beta version of the inventory with 385 questions, 99 of which were reverse scored. We administered this version of the survey to 10 staff members in randomized order through the SurveyMonkey platform.^1^ The staff highlighted questions that were unclear or not worded well, which were removed from the beta version. This resulted in a 175-item NSI beta version with approximately three items per construct and 24 reverse-scored items. We administered this 175-item NSI beta version anonymously using a randomized item order through SurveyMonkey to our recruited participants.

#### Statistical Analysis

All analyses were completed using the statistical programming language R v. 3.6.3 (R Development Core Team, 2021) using the package *psych* (Revelle, 2020). We conducted an EFA to determine factors that explained data variability. Iterative EFAs also allowed us to choose items that loaded more strongly than others on these factors, ultimately reducing the total item number for the NSI to below 50. Factors were estimated using parallel analysis and the unweighted least squares method since it carries no normality assumption (Flora et al., 2012) and with varimax rotation. The correlation matrix was inspected visually, looking for excessive correlations and multicollinearity and using the Kaiser, Meyer, and Olkin Measure of Sampling Adequacy (Revelle, 2020).

### Results

#### Participants

Nine hundred and eighty participants began the survey between 13 August 2020 and 16 November 2020. Five hundred and seven participants completed all items and were included in the analysis. Thirty-six participants were excluded for not meeting inclusion criteria (i.e., 1 < 18 years, 12 not fluent in English, and 23 had no noetic experience). Four hundred and thirty-two participants were excluded due to not completing the survey (i.e., 225 did not continue after the screening and consent questions, and 207 did not complete the entire survey).

#### Exploratory Factor Analysis

We examined the EFA results of the 175 items for high (≥ |0.5|) to moderate (between |0.2| and |0.5|) loadings. Items with loadings ≥ |0.5| for each factor and uniquely loaded (i.e., salient on only one factor with no complex or moderate to high cross-loadings) were retained. This process resulted in a 50-item scale (statistical output for this EFA is available upon request from the first author).

We then conducted another EFA of these 50-items, which resulted in an 11-factor solution. Four additional items were removed because they loaded moderately (|0.2|–|0.5|) on multiple factors. An EFA on the remaining 46-items resulted in an 11-factor solution with all 46-items loading ≥|0.48|. Statistical output and factor loadings of this EFA are displayed in Supplementary Tables 1 and 2, respectively. The 11 factors consisted of items relating to common noetic experience categories: (1) Inner Knowing (10-items), (2) Knowing Yourself (four-items), (3) Embodied Sensations (six-items), (4) Inner Voice and Apparent Communication with Non-physical Beings (five-items), (5) Inner Knowing Through Touch (three-items), (6) Visualizing to Access or Affect (four-items), (7) Healing (three-items), (8) Physical Sensations from Other People (three-items), (9) Knowing the Future (three-items), (10) Knowing Through Dreams (two-items), and (11) Knowing Other’s Minds (three-items).

### Study 1 Summary

Study 1 began with qualitative data from a previous study, creating a 385-item pilot version of the NSI, which was refined through internal testing to a 175-item version that was subsequently administered to 507 participants. EFA of this dataset resulted in an 11-factor solution explaining the data’s variability and 46-items that loaded well on those 11-factors. Limitations for Study 1 include the absence of participant demographics and a biased population since we recruited participants through the IONS membership.

## Study 2: NSI Convergent/Divergent Validity and Test–Retest Reliability

The purpose of this study was to assess the 11-factor model found in Study 1 using CFA of a different dataset, evaluate the convergent and divergent validity, and test–retest reliability.

### Methods

#### Participants

One thousand two hundred twenty-five participants were recruited from the IONS membership through e-newsletters, blogs, and an online recruitment posting. Inclusion criteria were: (1) age 18 years or older, (2) fluency in English, and (3) having had a prior noetic experience. All participants signed an informed consent to participate in the study consistent with the IONS Institutional Review Board guidelines. Participants received their NSI scores and a $400 gift card raffle entry.

For sample size selection, some sources suggest at least 10 people per item for psychometric validation, although a recent review suggested that sample size is rarely justified *a priori* (Anthoine et al., 2014). Similarly, there is no agreement on the number of participants needed for CFA, although some (Bentler, 1995) recommend approximately 10 participants for each estimated parameter. We aimed for a sample size of 500 to meet this general recommendation (10 × 46 parameters = 460).

#### Measures and Procedures

In order to evaluate the convergent/divergent validity of the model and the test–retest reliability of the established model in Study 1, two surveys were conducted. The first survey included demographic questions, the 46-item NSI from Study 1, and measures chosen to test convergent and divergent construct validity. The second survey consisted of the 46-item NSI only. We invited participants to complete this survey 3 weeks after the first administration to evaluate test–retest reliability.

Convergent construct validity measures included the anomalous/paranormal experience and the anomalous/paranormal ability subscales of the AEI (Gallagher et al., 1994) and the full NEBS (i.e., experience and belief subscales; Wahbeh et al., 2020b). These were the most closely matched for the goal of assessing convergent validity based on a review of measures in this domain (Wahbeh et al., 2020b). The Exceptional Experiences Questionnaire was not included because the AEI and NEBS adequately assessed convergent construct validity without adding the EEQ, which would add participant burden. We hypothesized that the NSI would have significant moderate correlations with these measures strengthening the interpretation of the noetic traits described in the NSI.

Divergent construct validity measures included the Big Five Inventory-10 (BFI-10; Rammstedt, 2007), the Short Profile of Emotional Competence (S-PEC; Mikolajczak et al., 2014), the Highly Sensitive Person Scale (HSPS; Aron and Aron, 1997a), the Community Assessment of Psychic Experiences—Positive Scale (CAPE-P15; Capra et al., 2013), and the Dissociation Experiences Scale Taxon (DES-T; Waller et al., 1996). The apparent variety in an individual’s noetic experiences and our proposal that each individual has a profile of noetic characteristics suggests that noetic characteristics may be an aspect of a person’s personality. However, we hypothesized that the NSI would not correlate with four of the five BFI-10 traits (i.e., extraversion, agreeableness, conscientiousness, and neuroticism) since noetic characteristics reflect extrasensory experiences which are not reflected by the big five traits. However, as noted above, characteristics measured by the BFI-10 trait openness are often predictive of noetic experiences and doing well on noetic tasks in the laboratory (Cardeña and Marcusson-Clavertz, 2015). Therefore, we hypothesized that the NSI might be slightly correlated with the openness trait on the BFI-10. We did not anticipate strong correlations since the NSI evaluates different constructs.

Similarly, many people who have noetic experiences score higher in empathy than those who do not (Irwin, 2017), and people with noetic experiences often score high on sensory processing sensitivity questionnaires. This type of sensitivity refers to a sensitivity to stimuli, deep processing of information, and more emotional and physiological reactivity (Aron and Aron, 1997b). The S-PEC and the HSPS were included to test correlations with measures of empathy and sensitivity. Again, we hypothesized that these measures would be only slightly correlated with the NSI, if at all, because while people who experience more noetic experiences may be more empathetic or sensitive, they are not the same constructs.

Finally, two mental health symptoms are often associated with noetic experiences: psychotic and dissociative. To test possible correlations with psychotic and dissociative measures, the CAPE-P15 and DES-T were included. We hypothesized that the NSI would not be highly correlated with either of these measures. Additional details on the convergent and divergent measures are included in the Supplementary Material.

#### Statistical Analysis

All data cleaning/organization and statistical analyses were conducted using R. The data were imported from an excel file using the *readxl* package (Wickham and Bryan, 2019). The dataset (with reverse coded items) was assessed for multivariate normality using the Mardia Skewness and Kurtosis multivariate normality (MVN) tests and Shapiro–Wilk univariate normality tests in the R package *MVN* (Korkmaz et al., 2014).

A standard CFA model was fit to the data using the R package *lavaan* (Rosseel, 2012) with the latent variable variance constrained to 1. The data were composed of 667 complete cases (no missing data). CFA was fit using the *nlminb* optimization method with an 11 latent variable model. Latent variable formulas are included in the Supplementary Material.

The data did not uphold the assumption of multivariate normality (Mardia Skewness Statistic = 49223.5, *p* = 0; Mardia Kurtosis Statistic = 156.99, *p* = 0, Univariate Shapiro Wilk all value of *p*’s <0.001). Therefore, the CFA was fit using the Maximum likelihood estimator with bootstrap method (1,000 replicates). Results and plots of the fitted models were generated using the R packages *semTable* (Johnson and Kite, 2020) and *lavaanPlot* (Lishinski, 2018). Composite measures were generated by predicting the data used to fit the CFA model. Subsequent EFAs were conducted in the same way as described in Study 1.

We calculated the means and standard deviations for all variables to assess validation. We evaluated Spearman’s rank-order (rho) correlation matrices for expected association patterns between measures of similar and different constructs. A Bonferroni multiple comparison correction was applied for the appropriate value of *p* cut-off for significant findings. As recommended, we assessed test–retest reliability with an Intraclass Correlation Coefficient (ICC; Aldridge et al., 2017).

### Results

#### Participants

One thousand two hundred twenty-five adults began the survey between 29 January 2021 and 22 April 2021. Six hundred and sixty-seven completed all NSI items and were included in the analyses resulting in a ratio of 14.5 participants to each parameter estimated. One hundred and twenty-one participants were excluded for not meeting inclusion criteria (i.e., 22 were not fluent in English, and 99 had no noetic experience). Two hundred and one participants were excluded for not signing the consent form, 234 did not complete the entire survey, and two people completed the survey twice.

Six hundred and thirty-four participants entered their demographic data (these fields were optional). Those who did were 56.5 ± 13.8 SD years old with 17.4 ± 3.6 SD years of education. There were 483 or 76% female, 142 or 22% male, and 9 or 1.4% of another gender. Participants could check multiple ethnicities that applied to them. The ethnic breakdown for those who answered the question was: Native American (41, 5.7%); Native Pacific Islander (7, 1.0%); Asian (36, 5.0%); African (29, 4.0%); Middle Eastern (21, 2.9%); Latinx or Hispanic (41, 5.7%); and European (545, 75.7%).

#### Refining the NSI and Confirming the Factor Model

We inadvertently duplicated one item and omitted one item in the survey. The omitted item had a factor loading of 0.53 on the Embodied Sensations factor and loaded between 0.21 and 0.27 on four other factors. Because the Embodied Sensations factor had five additional items in the inventory, we eliminated this item’s consideration from further inventory versions. Thus, we conducted the CFA on the 45 remaining items.

The CFA results for the 11-factor model were as follows: the model chi-square was 4189.0 (*p* < 0.00005), the root mean square error of approximation (RMSEA) was 0.07 (90% confidence interval 0.07–0.07), and the Tucker-Lewis fit index (TLI) was 0.80 (see Supplementary Table 3 for detailed statistical output). These RMSEA and TLI values represent a weak to good model fit to the dataset, as indicated by commonly reported fit statistics (Hooper et al., 2007; Parry, 2019).

Through reviewing the factor loadings of this analysis, another item from the Knowing Yourself factor was removed because it no longer loaded at a value greater than 0.50 on any factor, and the Knowing Yourself still had five remaining items. Thus, the NSI now had 44-items.

While the RMSEA was satisfactory for a good fit, the TLI score was not. In order to ensure a model with a good fit, we conducted another EFA on a combined dataset consisting of (1) the 44-item dataset from this study (Study 2) and (2) the corresponding 44-item subset of Study 1 175-item dataset (see Figure 1). That is, the CFA did not reveal a good fit for the 11-factor model, so we conducted another EFA with more data to evaluate the best model to fit the data. This new EFA resulted in a 12-factor model with better model indices, RMSEA = 0.038, 90% confidence intervals 0.036–0.041, and TLI = 0.943 (see Supplementary Table 4 for detailed statistical output and Supplementary Table 5 for factor loadings for each item of the 44-item NSI).

The 12-factors consisted of the same content categories as the 11-factors. However, they were in a different order, reflecting changes in the variability explained by each factor in the larger dataset. Interestingly, all the factors in the 12-factor model consisted of the same items as the 11-factor model except for the Inner Voice, Apparent Communication with Non-physical Beings factor, which was split into two different factors in the new 12-factor model: Inner Voice items were in one factor, and Apparent Communication with Non-physical Beings items were in a different factor. This division is more reflective of the reported subjective experiences of these two phenomena, with Inner Voice indicative of clairaudient experiences like the Socrates anecdote described in the introduction and Apparent Communication with Non-physical Beings items being indicative of widespread contact with the dead phenomena (Wahbeh et al., 2018b).

Thus, a final 44-item NSI was created with a 12-factor model solution through iterative testing and analyses. The content categories and order for the 12-factors were: (1) Inner Knowing, (2) Embodied Sensations, (3) Visualizing to Access or Affect, (4) Inner Knowing Through Touch, (5) Healing, (6) Knowing the Future, (7) Physical Sensations from Other People, (8) Knowing Yourself, (9) Knowing Other’s Minds, (10) Apparent Communication with Non-physical Beings, (11) Knowing Through Dreams, and (12) Inner Voice. Please contact the first author for the NSI’s items.

#### Construct Validity

Table 1 depicts the means, standard deviations, and Cronbach’s alphas for the global score and factors of the 44-item NSI. The Cronbach’s alpha for all 44-items was 0.94, and 12-factor alphas ranged from 0.76 to 0.94, demonstrating a high degree of internal consistency for the factor items and overall.

**Table 1:** Mean values, standard deviations, and Cronbach’s alphas for measures.

	Time 1 Mean ± SD n = 667	Cronbach’s *ɑ* n = 667	Time 2 Mean ± SD n = 542	Test–retest ICC [95% CI]^*^
**Noetic Signature Inventory**				
NSI Total	68.2 ± 14.5	0.94	67.0 ± 15.2	0.88 [0.86–0.90]
NSI1 Inner Knowing (10)	76.5 ± 15.9	0.86	75.3 ± 16.3	0.83 [0.80–0.86]
NSI2 Embodied Sensations (6)	42.9 ± 22.8	0.79	42.7 ± 22.3	0.83 [0.80–0.85]
NSI3 Visualizing to Access or Affect (4)	68.2 ± 23.7	0.83	67.9 ± 24.1	0.76 [0.73–0.80]
NSI4 Inner Knowing Through Touch (3)	46.0 ± 30.8	0.92	45.6 ± 31.2	0.82 [0.79–0.84]
NSI5 Healing (3)	64.3 ± 29.2	0.90	63.1 ± 29.1	0.88 [0.85–0.89]
NSI6 Knowing the Future (3)	75.9 ± 21.4	0.88	74.1 ± 20.5	0.82 [0.79–0.85]
NSI7 Physical Sensations from Other People (3)	61.2 ± 28.9	0.89	61.4 ± 27.3	0.84 [0.81–0.86]
NSI8 Knowing Yourself (4)	81.3 ± 16.6	0.76	79.6 ± 17.8	0.67 [0.62–0.72]
NSI9 Knowing Other’s Minds (3)	64.9 ± 23.9	0.80	62.2 ± 24.7	0.77 [0.73–0.80]
NSI10 Apparent Communication with Non-physical Beings (3)	72.1 ± 21.5	0.79	70.1 ± 21.9	0.81 [0.77–0.83]
NSI11 Knowing Through Dreams (2)	73.9 ± 26.7	0.92	72.4 ± 26.1	0.78 [0.75–0.81]
NSI12 Inner Voice (2)	74.4 ± 26.0	0.86	72.8 ± 25.8	0.73 [0.68–0.76]
**Convergent Validity**				
NEBS—Belief	88.6 ± 10.4	0.84		
NEBS—Experience	68.6 ± 16.7	0.83		
AEI—Anomalous/paranormal ability	7.2 ± 4.4	0.86		
AEI—Anomalous/paranormal experience	14.9 ± 6.7	0.87		
**Divergent Validity**				
BFI-10—Extraversion	3.0 ± 1.1	0.64		
BFI-10—Agreeableness	3.7 ± 0.9	0.29		
BFI-10—Conscientiousness	1.9 ± 0.9	0.45		
BFI-10—Neuroticism	3.4 ± 1.1	0.64		
BFI-10—Openness	1.9 ± 0.9	0.18		
CAPE—P15	5.0 ± 4.5	0.83		
S-PEC	3.5 ± 0.4	0.70		
DES-T	8.2 ± 11.5	0.82		
HSPS	36.5 ± 4.5	0.74		

* All value of *p*’s for ICCs are <0.000005.

NSI, Noetic Signature Inventory; NEBS, Noetic Experience and Belief Scale; AEI, Anomalous Experiences Inventory; BFI-10, Brief Five Inventory—10; CAPE-P15, Community Assessment of Psychic Experiences—Positive Scale; S-PEC, Short Profile of Emotional Competence; DES-T, Dissociation Experiences Scale Taxon; and HSPS, Highly Sensitive Person Scale.

Table 2 is a correlation matrix displaying the relationships between NSI factors and construct validity measures. Most NSI factor correlation pairs were positive and significant at the *p* < 0.00005 level. Significant correlations ranged from 0.18 to 0.54. Forty correlations had significant low correlation values (0.30–0.49), and five had significant moderate correlation values (>0.50).

**Table 2:** Correlation matrix for NSI Construct Validity.

	Measure	1	2	3	4	5	6	7	8	9	10	11	12	13	14	15	16	17	18	19	20	21	22	23	24	25	26
1	**NSI-1**	1																									
2	**NSI-2**	**0.39**	1																								
3	**NSI-3**	**0.31**	*0.19*	1																							
4	**NSI-4**	**0.38**	**0.47**	0.25	1																						
5	**NSI-5**	**0.43**	**0.33**	**0.43**	**0.41**	1																					
6	**NSI-6**	**0.52**	0.27	0.26	**0.31**	0.23	1																				
7	**NSI-7**	**0.54**	**0.47**	0.28	**0.47**	**0.50**	**0.34**	1																			
8	**NSI-8**	**0.37**	0.15	**0.38**	*0.19*	**0.33**	**0.31**	0.23	1																		
9	**NSI-9**	**0.53**	**0.38**	**0.34**	**0.38**	**0.40**	**0.47**	**0.46**	**0.33**	1																	
10	**NSI-10**	**0.48**	**0.37**	**0.44**	**0.41**	**0.42**	**0.36**	**0.40**	**0.43**	**0.48**	1																
11	**NSI-11**	0.27	0.16	**0.30**	0.11	*0.18*	**0.34**	*0.19*	0.28	0.27	0.29	1															
12	**NSI-12**	**0.43**	**0.30**	**0.39**	0.21	0.26	**0.32**	0.27	**0.44**	**0.39**	**0.50**	0.27	1														
13	**NSITotal**	**0.77**	**0.61**	**0.56**	**0.63**	**0.66**	**0.58**	**0.70**	**0.50**	**0.70**	**0.71**	**0.44**	**0.59**	1													
14	**NEBS-B**	**0.43**	0.23	**0.33**	*0.18*	0.28	**0.33**	0.26	**0.41**	**0.36**	**0.44**	0.30	**0.42**	**0.48**	1												
15	**NEBS-E**	**0.56**	**0.47**	**0.38**	**0.48**	**0.46**	**0.53**	**0.44**	**0.39**	**0.58**	**0.63**	**0.31**	**0.45**	**0.74**	**0.49**	1											
16	**AEI-Ab**	**0.47**	**0.43**	**0.43**	**0.49**	**0.42**	**0.40**	**0.48**	**0.30**	**0.51**	**0.62**	0.22	**0.37**	**0.70**	**0.36**	**0.69**	1										
17	**AEI-E**	**0.40**	**0.49**	**0.35**	**0.49**	0.29	**0.50**	**0.46**	0.26	**0.52**	**0.57**	**0.32**	**0.37**	**0.71**	**0.35**	**0.70**	**0.71**	1									
18	**E**	0.03	0.07	0.07	0.06	0.11	0.06	0.08	0.07	0.03	0.09	0.05	0.07	0.10	0.01	0.08	0.13	0.09	1								
19	**A**	0.02	−0.02	0.00	−0.04	0.06	0.01	0.03	0.07	0.02	0.05	0.02	0.05	0.02	0.08	0.08	0.00	−0.01	0.14	1							
20	**C**	−0.12	0.00	−0.06	−0.02	−0.08	−0.04	−0.03	−0.10	−0.07	−0.08	−0.03	−0.09	−0.09	−0.07	−0.11	−0.02	0.02	−0.1	−0.04	1						
21	**N**	0.04	0.00	0.08	0.06	0.11	0.03	0.01	0.14	0.03	0.07	0.00	0.07	0.07	0.02	0.09	0.10	−0.03	0.20	0.28	−0.12	1					
22	**O**	−0.12	−0.10	*−0.19*	−0.16	−0.09	−0.10	−0.10	−0.15	−0.15	−0.17	−0.21	−0.09	−0.21	−0.08	−0.15	−0.13	*−0.19*	−0.05	0.01	0.06	0.05	1				
23	**CAPE-P15**	0.22	**0.34**	0.08	0.20	0.11	0.21	0.24	0.08	0.23	0.20	0.06	0.12	**0.30**	0.15	0.22	0.25	**0.41**	−0.02	−0.12	0.14	−0.21	−0.04	1			
24	**S-PEC**	**0.36**	0.05	*0.19*	0.06	0.26	*0.19*	0.21	0.28	0.22	0.19	0.13	0.23	0.28	0.26	0.22	*0.19*	0.11	*0.18*	*0.18*	−0.21	0.26	−0.10	−0.16	1		
25	**DES-T**	0.13	0.28	0.02	0.10	0.07	0.11	0.11	0.05	0.13	0.09	0.00	0.15	*0.19*	0.08	0.15	0.17	0.29	−0.00	−0.06	0.13	−0.09	0.02	**0.61**	−0.13	1	
26	**HSPS**	**0.39**	*0.19*	0.26	0.21	0.21	0.22	0.26	0.27	0.27	0.22	0.22	0.24	**0.39**	0.29	**0.31**	0.21	0.27	0.04	0.09	*−0.19*	0.02	*−0.31*	0.06	**0.38**	0.08	1

Spearman’s rho correlation coefficients are displayed for each questionnaire and subscales. With a Bonferroni multiple comparison adjustment, a significant value of *p* would be <0.0021 (0.05/25*25/2–25). For correlations between NSI factors and convergent validity measures of Noetic Experience and Belief Scale and Anomalous Experiences Inventory Experience and Ability subscales, all *p*’s ≤ 0.000005. Shaded cells represent *p*’s ≤ 0.000005. Italics font represents *p* < 0.0021 and greater than >0.000005. Bold font represents Spearman’s rho values that have a low correlation (0.30–0.49), bold and underlined fonts represent values that are moderately correlated (0.50–0.69), bold and double-underlined fonts represent values that are highly correlated (0.70–0.89). No values were very highly correlated (0.90–1.0). Spearman’s rho values 0–0.29 were considered to be negligible. NSI, Noetic Signature Inventory; NEBS, Noetic Experience and Belief Scale; AEI, Anomalous Experiences Inventory; E, Extraversion; A, Agreeableness; C, Conscientiousness; N, Neuroticism; O, Openness; CAPE-P15, The Community Assessment of Psychic Experiences-Positive Scale; S-PEC, Short Profile of Emotional Competence; DES-T, Dissociation Experiences Scale Taxon; and HSPS, Highly Sensitive Person Scale. NSI Factor descriptions: (1) Inner Knowing, (2) Embodied Sensations, (3) Visualizing to Access or Affect, (4) Inner Knowing Through Touch, (5) Healing, (6) Knowing the Future, (7) Physical Sensations from Other People, (8) Knowing Yourself, (9) Knowing Other’s Minds, (10) Apparent Communication with Non-physical Beings, (11) Knowing Through Dreams, and (12) Inner Voice.

#### Convergent Validity

Spearman’s rho values were classified as follows: (1) negligible (<0.29), (2) low (0.30–0.49), (3) moderate (0.50–0.69), and (4) high (0.70–0.89). Both the AEI anomalous/paranormal experience subscale and anomalous/paranormal ability subscale were highly significant with the NSI Total (0.71 and 0.70, respectively). In addition, low to moderate significant correlations were found for all NSI factors with both AEI subscale scores except for three correlations that were only negligible. These negligible correlations were found for the AEI experience subscale and NSI factors 5 (i.e., Healing; 0.29) and 8 (i.e., Knowing Yourself; 0.26); and for the AEI ability subscale and NSI factor 11 (i.e., Knowing Through Dreams; 0.22). The NEBS experience subscale was highly correlated with the NSI Total (0.74), with low to moderate correlations found for the individual factors. In contrast, the NEBS belief subscale was lowly correlated with the NSI Total (0.48), with low correlations found for the majority of the individual factors, negligible correlation to factors 2, 5, and 7 (i.e., Embodied Sensations, Healing, and Physical Sensations from Other People), and no significant correlation to factor 4 (i.e., Inner Knowing Through Touch).

#### Divergent Validity

As for convergent validity, Spearman’s rho values were classified as follows: (1) negligible (<0.29), (2) low (0.30–0.49), (3) moderate (0.50–0.69), and (4) high (0.70–0.89). The NSI Total and none of the NSI factors were correlated with the personality traits of Extraversion, Agreeableness, Conscientiousness, and Neuroticism. Openness had a significant negligible correlation with NSI factor 3 (i.e., Visualizing to Access or Affect), 11 (i.e., Knowing Through Dreams), and NSI Total (−0.19, −0.21, and −0.21, respectively). The S-PEC had a significant low correlation with factor 1 (i.e., Inner Knowing; 0.36). In addition, the S-PEC had significant negligible correlations with factors 5 (i.e., Healing; 0.26), 7 (i.e., Physical Sensations from Other People; 0.21), 8 (i.e., Knowing Yourself; 0.28), 9 (i.e., Knowing Other’s Minds; 0.22), 12 (i.e., Inner Voice; 0.23), and the Total NSI (0.28). The HSPS had significant low correlations with factor 1 (i.e., Inner Knowing; 0.39) and the NSI Total (0.39). All other factors had negligible correlations, except for factor 2 (i.e., Embodied Sensations), which was not significantly correlated. The CAPE-P15 for psychotic symptoms had significant low correlations with factor 2 (i.e., Embodied Sensations; 0.34) and NSI Total (0.30). Further, negligible correlations were found with factors 1 (i.e., Inner Knowing; 0.22), 4 (i.e., Inner Knowing Through Touch; 0.20), 6 (i.e., Knowing the Future; 0.21), 7 (i.e., Physical Sensations from Other People; 0.24), 9 (i.e., Knowing Other’s Minds; 0.23), and 10 (i.e., Apparent Communication with Non-physical Beings; 0.20). The DES-T for dissociative symptoms had a significant negligible correlation with NSI factor 2 (i.e., Embodied Sensations; 0.28). No other correlations were significant for the DES-T.

#### Test–Retest Reliability

Five hundred and forty-two participants completed the second administration of the NSI between 18 February 2021 and 20 May 2021. The average number of days between administrations was 26.0 ± 12.5 days. The means and standard deviations are displayed in Table 1, along with ICCs estimate correlations between individual measurements for test–retest reliability. The two administrations were highly correlated (0.67–0.88 for 12 factors; 0.88 NSI overall).

### Study 2 Summary

Study 2 began with a 46-item NSI with an 11-factor model. Through an iterative process of factor analyses, a final 44-item NSI was developed with a 12-factor model. The 12-factor model had strong model indices and better reflected subjective experiences of the noetic with two different phenomena that were previously combined into one factor were separated into two factors. The 44-item NSI demonstrated high internal consistency.

In terms of convergent validity, as hypothesized, the NSI Total score was highly correlated with the AEI anomalous/paranormal experience and anomalous/paranormal ability subscales. In addition, low to moderate significant correlations were found for most individual NSI factors and the AEI subscale scores. The AEI items are the most similar in concept to what the NSI attempts to evaluate. Thus, these results strengthen the interpretation of the noetic traits described in the NSI. However, the AEI does not clearly explore the nuanced way in which an individual receives and experiences noetic information to provide a personal profile of noetic characteristics. In addition, many items have biased language. For example, one question is “I am psychic,” which is problematic since psychic may mean different things to different people. The NSI Total score was also found to be highly correlated with the NEBS experience subscale with low to moderate correlations for the individual factors. These results again support the interpretation of the noetic traits described in the NSI. The correlation with the NEBS belief subscale was low to moderate for the individual factors. This makes sense as the experience subscale would more clearly assess the subjective personal expression of accessing inner wisdom as opposed to a belief in these experiences. This reflects some overlap between experience and belief that has also been seen in other studies (Glicksohn, 1990; Spinelli et al., 2002; Wahbeh et al., 2018b, 2020b).

In terms of divergent validity, as hypothesized, the NSI Total score and individual scores did not significantly correlate with the personality traits of Extraversion, Agreeableness, Conscientiousness, and Neuroticism. As hypothesized, there was a correlation to openness, but this was negligible. In addition, this correlation was negative, which was unexpected but still confirms our hypothesis that the NSI factors are distinct from commonly measured personality traits. Similarly, the S-PEC and HSPS had negligible correlations with most NSI factors. However, both the S-PEC and HSPS had low correlations with factor 1 (i.e., Inner Knowing). This suggests that the noetic experience of inner knowing or intuition may be associated with the personality traits of empathy and sensitivity. For psychotic and dissociative symptoms, as expected, the NSI was not highly correlated with either the CAPE-P15 or the DEST-T. Interestingly, both measures were slightly correlated with the NSI factor 2 (i.e., Embodied Sensations), a factor that may be associated with such experiences as hearing voices or disembodied sensations (Aron and Aron, 1997b; Lewandowski et al., 2009; Moreira-Almeida and Cardeña, 2011; Roxburgh and Roe, 2011; Irwin, 2017; Sagher et al., 2019; Wahbeh et al., 2020b; Pederzoli et al., 2021). After 3 weeks, the 44-item NSI test–retest reliability evaluation showed high consistency. Likely, the NSI factors represent traits rather than a state, but further research is required to assess this more definitively. Thus, the 44-item NSI with a 12-factor model demonstrated high internal consistency, expected construct validity, and strong test–retest reliability.

## Study 3: Confirming NSI 12-Factor Model

The purpose of Study 3 was to confirm the 12-factor model of the 44-item NSI in a more diverse and independent sample.

### Methods

#### Participants

Two thousand eight hundred and twenty-seven participants were recruited through Lucid, LLC (New Orleans, Louisiana). Lucid collects data directly from targeted audiences through surveys and cross-media measurement. Inclusion criteria were: (1) age 18 years or older, (2) fluency in English, and (3) having had a prior noetic experience. All participants signed an informed consent to participate in the study consistent with the IONS Institutional Review Board guidelines. The participants were compensated directly through the affiliate marketing contractor Lucid partnered with to obtain survey participants. Participants received approximately $1–3 to complete the survey (the payment amount is approximate because Lucid’s suppliers paid at different rates).

#### Measures and Procedures

Eligible participants were routed through the Lucid platform to the SurveyMonkey survey to complete the 44-item NSI with items displayed in a randomized order.

#### Statistical Analysis

All data cleaning/organization and statistical analyses were conducted using R. Data were imported from excel using the *readxl* package. We performed a data quality check of the participants’ data to ensure valid responses by evaluating the scores of the items with reverse-coded pairs (i.e., items 6, 25, 41, 42, and 44). For example, one item asks about receiving noetic information in dreams was paired with a reverse-coded item stating that they have not received any information through dreams. A participant who was thoughtfully answering the items would have opposite scores for these two items. The quality evaluation method resulted in 1,012 valid participant records that were included in the analysis. Regarding the appropriate sample size, 1,012 participant records reflected a ratio of 23 participants to each parameter estimated. See Supplementary Material for detailed methods on the data quality control process.

The updated dataset (with reverse coded items) was assessed for multivariate normality using the Mardia Skewness and Kurtosis multivariate normality tests and Shapiro–Wilk univariate normality tests in the R package *MVN* (Korkmaz et al., 2014).

A standard CFA model was fit to the data using the R package *lavaan* with the latent variable variance constrained to 1. The data were composed of 1,012 complete cases (no missing data). Given that the data did not uphold the assumption of multivariate normality, CFAs were fit using the diagonally weighted least squares (DWLS) estimator and the limited-memory Broyden–Fletcher–Goldfarb–Shanno optimization method (Byrd et al., 1995). The DWLS estimator was used for this CFA rather than the ML with bootstrap as previously used, as it was deemed a more appropriate estimator for this data because 0 and 100 data can be considered ordinal or numerical. That is, it does not quite fit ordinal data because there are so many categories, but they do not quite fit numerical data because they do not live on the real number line but an interval. It took some research and iteration to get to DWLS being an appropriate estimator and further iteration to find an optimizer that worked. The model was fit using 12 latent variables (see Supplementary Data for latent variable formulas). Results for the CFA were generated using the R package *semTable*.

### Results

#### Participants

Two thousand eight hundred twenty-seven volunteers started the survey between 15 June 2021 and 15 September 2021. One thousand seven hundred seventy-one participants completed all NSI items and were included in the analyses. Eight hundred twenty-nine participants were excluded for not meeting inclusion criteria (i.e., 106 < 18 years, 40 not fluent in English, and 683 had no noetic experience). Fifty-five participants were excluded for not signing the consent form, and 172 did not complete the entire survey. As noted above, examination of these records to ensure the inclusion of only valid data resulted in 1,012 records contributing to the CFA.

Most participants were from the United States (996, 98%), with 16 participants from other countries around the world (one participant each from Angola, Antigua and Barbuda, Argentina, Australia, Belize, Bhutan, Colombia, El Salvador, Ireland, Jamaica, Jordan, Nicaragua, Saint Lucia, South Africa, and two from Georgia). Participants were 45.2 ± 16.6 SD years old with 15.6 ± 2.8 SD years of education. Participant gender identifications were 588 or 58.8% female, 413 or 41% male, and 5 or 0.50% of another gender. Participants could check multiple ethnic categories that applied to them. The ethnic breakdown for those who answered the question was: Native American (228, 20.2%); Native Pacific Islander (18, 1.6%); Asian (43, 3.8%); African (93, 8.2%); Middle Eastern (18, 1.6%); Latinx or Hispanic (121, 10.7%); and European (607, 53.8%).

#### Confirmatory Factor Analysis

The CFA for the 12-factor model had a model chi-square of 1800.4 (*p* < 0.00005), RMSEA of 0.03 (90% confidence interval 0.03–0.04), and TLI of 0.99 (see Supplementary Table 6 for detailed statistical output). These values represent a good model fit (Hooper et al., 2007; Parry, 2019).

### Study 3 Summary

Study 3 confirmed the 12-factor model solution of the 44-item NSI in a more diverse participant population. The participants in Study 3 had greater ethnic diversity, were younger, and had less education than in Study 1 and 2, thus, reflecting a more generalizable population.

However, rigorous quality control measures had to be implemented to obtain a legitimate dataset. We recruited Studies 1 and 2 participants from IONS’ highly motivated members familiar with noetic experience topics. Response quality was dramatically reduced in the participants who were paid for their survey completion through Lucid. We had to collect 1.75 times more responses to achieve our final number. Also, while this method is highly accurate, there was still a chance of error, and we could expect that some small fraction of non-valid responses were classified as valid and vice versa. However, we expect this effect was negligible. Despite this extra data processing step, the final dataset from a diverse population enabled the 12-factor model solution CFA for the 44-item NSI.

Thus, the NSI can be considered a useful tool for tracking noetic characteristics within an individual (e.g., by re-taking the NSI at regular intervals over time) and assessing noetic characteristics between individuals (e.g., by having groups or populations take the NSI). Because there was inherent bias introduced by the data cleaning procedures for this study, additional studies to validate the NSI in other populations are warranted.

## General Discussion

This project’s goal was to develop the NSI, a self-report questionnaire that evaluates people’s noetic signatures with less than 50-items for ease of administration. We developed a 44-item inventory that assesses people’s noetic experience characteristics through an iterative qualitative and statistical process. The NSI demonstrated internal consistency, convergent and divergent content validity, and test–retest reliability. Also, the NSI’s 12-factor structure was confirmed in a diverse population.

### The 12-NSI Factors

The 12-factors that emerged through the factor analyses represent very distinct noetic experiences. These noetic experience categories have been studied and discussed for over 150 years (Cardeña et al., 2015), resulting in various levels of objective evidence for their validity. Despite the lack of extensive scientific research into these phenomena, people’s subjective experiences of them are commonplace and meaningful (Wahbeh et al., 2018b; Sagher et al., 2019). They also appear ubiquitous and have been described globally, with humanity’s oldest writings recording such experiences in cultures worldwide despite their ineffable nature. We include a brief description of each factor below and references for research examples where available. The descriptions below and supporting references are not meant to be comprehensive because a full review of these phenomena is beyond the scope of this paper, but a starting place for the reader to explore these subjective phenomena.

#### Factor 1: Inner Knowing

Inner Knowing represents general intuitive knowledge. It refers to the ability to “just know” something is true about people, places, or situations that could not be known or inferred by rational thought. As William James noted, this state of understanding comes with a feeling of authority on the knowledge (James, 1985). A typical participant statement was, “I just know it.” Numerous controlled experiments have explored the nature of general intuitive knowing (Schwartz, 2010; Radin, 2013; Cardeña et al., 2015; Wahbeh et al., 2022).

#### Factor 2: Embodied Sensations

Embodied Sensations refer to particular sensations, like heat or cold, goosebumps, smells, visions, tastes, sounds, dizziness, or tingles/vibrations/electricity, alerting the person that they are accessing noetic information. The body as a *receiver* or *sensor* of noetic information is well-known and studied (Radin and Pierce, 2015), with meta-analyses of independently conducted experiments demonstrating significant and replicable results (Schmidt et al., 2004; Schmidt, 2012, 2015). Multiple studies of one experimental paradigm where one person (the sender) alternates between sending and not sending their focused, positive intention toward another distant person (the receiver) have shown differences in the receiver’s physiology between the two conditions in a variety of multiple physiological measures, such as fMRI (Achterberg et al., 2005) and EEG (Richards et al., 2005).

#### Factor 3: Visualizing to Access or Affect

Visualizing to Access or Affect encompasses visualization or mental imagery to access information that the person would not usually know through their five senses or to affect the physical world. Research has shown that human intention can increase the probability of specific desired outcomes in the physical world. Examples include random number generator output (Schmidt, 2012) and plant growth (Shiah et al., 2017). The nuances of these mind-matter interactions have been explored (Schmidt, 1987; Kennedy, 1995; Radin, 2006), with much still to learn about how and why mind-matter interactions work. Researchers have also studied mental imagery as a way to access information for over 150 years. This phenomenon has recently received popular attention because of the Star Gate government project (May and Marwaha, 2018a) and demonstrates some of the most robust and reliable effects of all the noetic experiences (Baptista et al., 2015; Targ, 2019). There are practical applications and examples of this factor, such as predicting the stock market, futures or other financial market information, sports event outcomes, locations of missing persons or criminal cases, and finding unknown archeological sites (Harary and Targ, 1985; Kolodziejzyk, 2013; Schwartz, 2019).

#### Factor 4: Inner Knowing Through Touch

Inner Knowing Through Touch refers to a process called psychometry, where a person can touch an object and gain knowledge from it other than what one would usually know from their five senses (Barrington, 2016). While researchers studied psychometry in the late 1800s and early 1900s (Roll, 2004), little research has been conducted recently (Baker et al., 2017).

#### Factor 5: Healing

Healing represents the beneficial effects of positive intention. Numerous experiments demonstrate the significant positive effects found when people direct positive healing intention at humans, animals, plants, and cells (Roe et al., 2015). Energy medicine modalities, like Therapeutic Touch and Reiki, are also encompassed in this factor and have increasing objective evidence for their beneficial effects on conditions like pain, cancer, mental health symptoms, and hypertension (Jain et al., 2015; Rao et al., 2016; Yount et al., 2021). While the effects of distant intention are often small (0.10–0.25), considering that the effect should be zero, these results are intriguing.

#### Factor 6: Knowing the Future

Knowing the Future has been demonstrated in the laboratory while individuals are both conscious (Storm et al., 2012; Honorton et al., 2018) and unconscious, such as during sleep (Mossbridge and Radin, 2018; Storm and Tressoldi, 2020), as well as in everyday life (Dossey, 2009).

#### Factor 7: Physical Sensations From Other People

Physical Sensations from Other People, also known as telosomatic experiences, entail physical symptoms that people share at a distance. Clinicians and researchers have documented telesomatic experiences, but little research exists on them (Schwarz, 1973; Mann and Jaye, 2007; Dossey, 2016).

#### Factor 8: Knowing Yourself

Knowing Yourself reflects that noetic information supports personal growth, perception of oneself, and decision-making. Many studies have explored the positive impact noetic experiences can have on people’s lives, supporting them in being highly functional, well-adjusted with increased quality of life (Ellison and Fan, 2008; Moreira-Almeida and Cardeña, 2011; Sagher et al., 2019; Wahbeh and Butzer, 2020).

#### Factor 9: Knowing Other’s Minds

Knowing Other’s Minds represents mind-to-mind communication, which has objective and replicable evidence from multiple laboratory studies (Storm et al., 2010; Baptista et al., 2015; Cardeña, 2018; Storm and Tressoldi, 2020).

#### Factor 10: Apparent Communication With Non-physical Beings

Apparent Communication with Non-physical Beings refers to experiences like perceived contact with the dead. Perceived contact with the dead is a prevalent, widespread phenomenon, with 25%–53% of surveyed individuals in global studies reporting that they have had contact with the dead (Greeley, 1987; Haraldsson and Houtkooper, 1991; Pew Research Center, 2009). Triple-blind laboratory studies have demonstrated that professional mediums can obtain verifiably correct information about deceased people that they could not have known through traditional means (Beischel et al., 2015; Delorme et al., 2018).

#### Factor 11: Knowing Through Dreams

Knowing Through Dreams refers to accessing information through dreams that one would not usually know through traditional means. Decades of laboratory studies have demonstrated verifiable evidence of this phenomenon (Storm and Rock, 2015; Storm et al., 2017).

#### Factor 12: Inner Voice

Inner Voice refers to internal voice-hearing experiences that provide information not usually accessible through traditional means. A resurgence of interest in these experiences has recently arisen. Mental health researchers highlight that inner voice experiences are not always signs of mental illness but can be normal, functional, and add value to people’s lives (Powers et al., 2017; Richardson, 2018; Lee, 2020).

These 12 factors represent a wide variety of personal experiences of the noetic. Most also encompass the subjective experience of expanding beyond the personal self. We still do not understand the origin of these phenomena. Are they aspects of the unconscious mind with extended perception percolating to the surface? Perhaps they are aspects of the higher unconscious or superconscious, as Assogioli proposed, that individuals can access in different ways. Jung’s concept of the collective unconscious provides a container for noetic experiences in that it allows for interconnectedness among all humans. Accessing information beyond one’s usual knowledge through some communication with the collective unconscious is possible. Regardless, these experiences extend beyond the personal self and are transpersonal in nature and can be utilized in everyday life, through altered states of consciousness, like holotropic breathing, and in the laboratory, as presented in the section “Introduction.” Despite the apparent lack of understanding of how noetic experiences originate or operate, the process of developing and validating the NSI is just the beginning exploration of these transcendent and ubiquitous experiences.

## Limitations

There are several limitations to the studies that should be considered when considering the results. Future studies will incorporate remedies to address these limitations in the continued validation of the NSI. Study 1 failed to collect participant demographics which limits our understanding of generalizability to the general population. Study 1 and 2 recruited participants from the IONS membership, again limiting generalizability. However, the demographics for Study 2’s participants reflect other studies exploring similar topics (Wahbeh et al., 2018b). In an effort to increase generalizability, Study 3 engaged a recruitment firm to target participants with more diverse demographic characteristics. While this goal was achieved, it came at the cost of receiving invalid responses to the questionnaire (i.e., paired reverse-worded items were not consistent). Future studies would benefit from methods to ensure valid respondents prior to data collection. For example, could additional screening questions or within-questionnaire checks prevent invalid responses? Perhaps paired items that do not align could be flagged while the participant took the inventory and blocked them from continuing with invalid data, with an opportunity to correct their answer. These preventative measures would allow broad recruitment methods supporting diverse participants and generalizability while prevent invalid responses.

Another aspect related to the generalizability of the results was that most participants were from the United States. Future research should continue to assess participant demographics and their relationship to the noetic signature, especially with the administration to other English-speaking people worldwide. Subsequent translations into other languages would support the continued research into ubiquitous noetic phenomena globally.

For convergent and divergent validity, several different instruments could have been chosen. Future studies may include correlative studies with other perhaps related constructs. For example, boundary thinness is characterized by openness, sensitivity, and shifting between states of consciousness and has been related to noetic experiences like communication with apparent non-physical beings (Roxburgh and Roe, 2011, p. 281). Also, transliminality refers to the movement of material from thresholds of our consciousness, such as from the subliminal to supraliminal (Lange et al., 2000), and has been associated with noetic experiences (Houran and Lange, 2009). Additional research exploring other constructs related to noetic phenomena will support understanding the noetic signature’s relationship to them.

The statistical approach taken for the study including EFAs followed by CFA on an independent sample, which is a standard procedure. Future studies can consider other methodologies, such as bifactor analysis, exploring how robust the NSI data is to different types of CFAs (Jennrich and Bentler, 2011; Reise, 2012).

## Future Directions

Besides future directions to address the limitations of these studies, subsequent research using the NSI will support the elucidation of the noetic signature intra- and inter-variation, the prevalence of the 12 factors, their interactions with each other, if any, and potentially beneficial applications of the information the NSI provides. As described in the section “Introduction,” many studies evaluate the prevalence of noetic experiences. The NSI will support this effort by contributing additional nuance to the various noetic experience types. Another interesting research area will be the potential patterns in NSI factor expression. Are there factors that naturally group together? For example, perhaps people who score high on the Embodied Sensations factor also score high on the Physical Sensations from Other People factor. There are glimpses of these patterns from the correlations conducted, such as the moderate correlation between the Healing factor and Physical Sensations from Other People factor or Apparent Communication with Non-physical Beings and Inner Voice. However, more sophisticated analyses with larger datasets would support the revelation of any complex patterns in noetic signature expression.

Further, many have wondered if there is a biological underpinning to noetic experiences. Some preliminary studies and extensive subjective reports show that specific noetic experiences run in families (Neppe and Hurst, 1981; Cohn, 1994, 1999; Wahbeh et al., 2020a). Is there a genetic influence on a person’s noetic signature? Additional research can build on these preliminary genetic studies using the NSI. Ongoing studies are also examining brain structure and noetic experiences *via* structural neuroimaging data (Pantazatos, 2022).

Another critical aspect of the noetic signature to pursue is the extent to which it is changeable. In Study 2, test re-test reliability was validated but only at 3 weeks. If participants took the NSI in 1 year, would their scores still be highly correlated? We speculate that the noetic signature has a baseline structure but is malleable to some fluctuation. Perhaps genetic factors contribute to one’s innate noetic signature, but environmental factors can shift the noetic signature’s expression. For example, one individual may have a low score on the Visualizing to Access or Affect, including remote viewing experiences. They then take a course through the International Remote Viewing Association, where numerous courses are taught to cultivate such skills. Many teachers of these courses believe that anyone can learn how to remote view and that training and practice can improve one’s ability. The United States military capitalized on precisely this concept when they trained naïve soldiers in remote viewing (Hubbard and Langford, 1986). After taking such courses and extensive practice, would the person who previously scored low on the Visualizing to Access or Affect factor increase their score? The NSI may help us to distinguish between innate capacities that likely influence ability (i.e., will they do well at various laboratory tasks without training or practice) and skill that can be developed through training and practice. These are research questions that can be answered through subsequent research.

Research questions arise about how the noetic signature may impact and inform various domains of personal growth. For example, do successful business leaders have specific noetic signatures, like Inner Knowing? Are those different than professions that benefit from Embodied Knowing, such as fighter pilots, who often react viscerally in high-speed situations? Also, noetic experiences are intrinsically transcendent, and thus, their study will inform transpersonal psychology and our experience and relationships to our transcendent selves. With the upsurge in psychedelic medicine research (Mitchell et al., 2021), the NSI can be a valuable tool to explore the characteristics of ones’ noetic signature as a result of altered states of consciousness. Could knowing one’s noetic signature support them in learning situations like understanding one’s learning style (e.g., visual, auditory, and kinesthetic) aides students in studying technique? Not that one’s noetic signature should be viewed as a constrained limitation to one’s choices or expression, but it could facilitate self-awareness in various domains (e.g., personal development, education, career counseling, and clinical therapies).

A vital reason to continue research on the noetic signature is its relevance to positive psychology, especially well-being and meaning in one’s life. People find their many noetic experiences beneficial, inspirational, and positively impactful in their lives (Richards, 1991; Kennedy and Kanthamani, 1995a; Ellison and Fan, 2008; Griffiths et al., 2008; Wahbeh et al., 2018b). For example, one study found that people’s belief in life after death and a guiding or protective higher force increased after their experiences, as did their interest in spirituality, sense of connection to others, happiness, well-being, confidence, optimism about the future, and meaning in life (Kennedy and Kanthamani, 1995a). Furthermore, their fear of death, depression or anxiety, isolation and loneliness, and worries and fears about the future decreased. This study highlights that noetic experiences can be positively impactful, meaningful, and integrative. Studies that examine the more rare experiences of apparent communication with non-physical beings find benefits and positive impacts from them, including improved quality of life, greater meaning (Negro et al., 2002; Moreira-Almeida and Cardeña, 2011; Wahbeh et al., 2018a, 2019; Wahbeh and Butzer, 2020) and even grief resolution after the death of loved ones (Beischel, 2019).

Perhaps most importantly to the individual level, the NSI supports the normalization of these common experiences that are, for the most part, taboo in Western culture. Noetic experiences are gaining increased acceptance in many areas, yet skepticism and intense criticism for even bringing up the topics are intense (Cardeña, 2015). Many clinicians have highlighted the importance of depathologizing noetic experiences in high functioning individuals (Hearing Voices Network: Welcome, n.d.; Home | Yale COPE Project, n.d.; Pederzoli et al., 2021), inspiring the integrative, meaning, and improved quality of life the phenomena often bring (Sagher et al., 2019). Clinicians could use the NSI as a tool for exploration with their clients in a supportive container of the therapeutic relationship. There is often a sense of relief when individuals realize that they will be heard and taken seriously without being pathologized for sharing such experiences (Wahbeh et al., 2022). We anticipate that safe havens for open discussion about noetic phenomena will only strengthen our understanding of them and their usefulness to the human experience regardless of their objective validity. As Dr. Grof states, “More and more people seem to realize that genuine spirituality based on profound personal experience is a vitally important dimension of life” (Grof, 2019, 314). Like Drs. Freud, Young, James, and many others before Dr. Grof propose that human experiences beyond the personal self, while perhaps taboo to explore, are worthy of our attention and rigorous examination.

## Conclusion

The 12 factors represent noetic experiences that extend beyond our traditional five senses. One might call them extended perceptions. While many of these phenomena do not have overwhelming evidence of their objective veracity, and we still do not fully understand how these experiences might work, as we saw in Socrates’ anecdote, their benefit to the individual is clear. Our next step is to use the NSI to evaluate the nuances of these experiences within and across individuals, enhancing our understanding of their unique expression in humanity.

## Data Availability Statement

The datasets presented in this study can be found in online repositories. The names of the repository/repositories and accession number(s) can be found at: 10.6084/m9.figshare.17029883.

## Ethics Statement

All study activities were approved and overseen by the Institutional Review Board at the Institute of Noetic Sciences (IORG#0003743). The participants provided their written informed consent to participate in this study.

## Author Contributions

HW contributed to the conceptualization, funding acquisition, methodology, formal analysis, investigation, data curation, project administration, writing—original draft, and writing—review and editing. NF and PS contributed to the conceptualization, methodology, formal analysis, and writing—review and editing. All authors contributed to the article and approved the submitted version.

## Funding

This work was supported by the John Brockway Huntington Foundation, the Patricia Beck Phillips Foundation, and the John Sperling Foundation.

## Conflict of Interest

The authors declare that the research was conducted in the absence of any commercial or financial relationships that could be construed as a potential conflict of interest.

## Publisher’s Note

All claims expressed in this article are solely those of the authors and do not necessarily represent those of their affiliated organizations, or those of the publisher, the editors and the reviewers. Any product that may be evaluated in this article, or claim that may be made by its manufacturer, is not guaranteed or endorsed by the publisher.

## Footnotes

^1^ www.surveymonkey.com
