# Supplementary material for: The Noetic Signature Inventory: Development, Exploration, and Initial Validation
Source: Front Psychol. 2022 Jun 15;13:838582. doi: 10.3389/fpsyg.2022.838582 (PMC9240696; doi:10.3389/fpsyg.2022.838582)
Supplement: Supplementary file 1 [file Data_Sheet_1.docx]

Supplementary Material

[**STUDY 1**](#Study1)

[Table 1. 46-item NSI EFA statistical output](#Table1_46statsoutput)

[Table 2. Factor Loadings for each item of the 46-item NSI](#Table2_46_FactorLoadings)

[Table 3. Eigenvalues for 46-item NSI](#eigen46item)

[**STUDY 2**](#Study2)

[Measures](#Study2Measures)

[Construct Validity Measures](#ConstructValidity)

[Convergent Validity](#ConvergentValidity)

[Divergent Validity](#DivergentValidity)

[11-Factor Model CFA Formulas for 46-item NSI](#CFA11factor46item)

[Table 3. 11-Factor CFA fitted model](#Table3_11factorCFA)

[Table 4. Statistical output of EFA with larger dataset](#Table4_EFAlargedataset)

[12-Factor Model Formulas for 44-item NSI](#Formula12factor44item)

[Table 5. Factor Loadings for each item of the 44-item NSI](#Table5_Factorloadings44)

[Table 6. Eigenvalues for 44-item NSI](#eigen44item)

[**STUDY 3**](#Study3)

[Data Quality Control](#DataQualityControl)

[Table 6. CFA fitted model using diagonally weighted least squares](#Table6_CFAstatsoutput)

[**Final NSI Question Categories**](#FinalNSI)

[**REFERENCES**](#References)

## STUDY 1

**Table 1. 46-item NSI EFA statistical output**

|  | SS loadings | Proportion Var | Cumulative Var | Proportion Explained | Cumulative Proportion |
| --- | --- | --- | --- | --- | --- |
| 1 | 5.52 | 0.12 | 0.12 | 0.2 | 0.2 |
| 7 | 2.94 | 0.06 | 0.18 | 0.11 | 0.3 |
| 6 | 2.7 | 0.06 | 0.24 | 0.1 | 0.4 |
| 11 | 2.43 | 0.05 | 0.3 | 0.09 | 0.49 |
| 2 | 2.41 | 0.05 | 0.35 | 0.09 | 0.57 |
| 3 | 2.34 | 0.05 | 0.4 | 0.08 | 0.66 |
| 9 | 2.18 | 0.05 | 0.45 | 0.08 | 0.73 |
| 4 | 2.13 | 0.05 | 0.49 | 0.08 | 0.81 |
| 5 | 2.12 | 0.05 | 0.54 | 0.08 | 0.89 |
| 8 | 1.69 | 0.04 | 0.58 | 0.06 | 0.95 |
| 10 | 1.52 | 0.03 | 0.61 | 0.05 | 1 |

**Table 2. Factor Loadings for each item of the 46-item NSI.**

| **NSI Q #** | **1** | **2** | **3** | **4** | **5** | **6** | **7** | **8** | **9** | **10** | **11** |
| --- | --- | --- | --- | --- | --- | --- | --- | --- | --- | --- | --- |
| 25 | 0.51 |  |  |  |  |  |  |  |  |  |  |
| 35 | 0.56 |  |  |  |  |  |  |  |  |  |  |
| 15 | 0.57 |  |  |  |  |  |  |  |  |  |  |
| 13 | 0.60 |  |  |  |  |  |  |  |  |  |  |
| 12 | 0.63 |  |  |  |  |  |  |  |  |  |  |
| 27 | 0.66 |  |  |  |  |  |  |  |  |  |  |
| 5 | 0.67 |  |  |  |  |  |  |  |  |  |  |
| 11 | 0.67 |  |  |  |  |  |  |  |  |  |  |
| 4 | 0.68 |  |  |  |  |  |  |  |  |  |  |
| 23 | 0.70 |  |  |  |  |  |  |  |  |  |  |
| 43 |  | 0.56 |  |  |  |  |  |  |  |  |  |
| 19 |  | 0.61 |  |  |  |  |  |  |  |  |  |
| 18 |  | 0.68 |  |  |  |  |  |  |  |  |  |
| 10 |  | 0.72 |  |  |  |  |  |  |  |  |  |
| 40 |  |  | 0.52 |  |  |  |  |  |  |  |  |
| 7 |  |  | 0.53 |  |  |  |  |  |  |  |  |
| 30 |  |  | 0.57 |  |  |  |  |  |  |  |  |
| 41 |  |  | 0.58 |  |  |  |  |  |  |  |  |
| 29 |  |  | 0.59 |  |  |  |  |  |  |  |  |
| 1 |  |  | 0.61 |  |  |  |  |  |  |  |  |
| 22 |  |  |  | 0.48 |  |  |  |  |  |  |  |
| 16 |  |  |  | 0.52 |  |  |  |  |  |  |  |
| 44 |  |  |  | 0.62 |  |  |  |  |  |  |  |
| 21 |  |  |  | 0.70 |  |  |  |  |  |  |  |
| 36 |  |  |  | 0.70 |  |  |  |  |  |  |  |
| 46 |  |  |  |  | 0.61 |  |  |  |  |  |  |
| 38 |  |  |  |  | 0.75 |  |  |  |  |  |  |
| 31 |  |  |  |  | 0.82 |  |  |  |  |  |  |
| 20 |  |  |  |  |  | 0.48 |  |  |  |  |  |
| 37 |  |  |  |  |  | 0.57 |  |  |  |  |  |
| 6 |  |  |  |  |  | 0.63 |  |  |  |  |  |
| 33 |  |  |  |  |  | 0.81 |  |  |  |  |  |
| 9 |  |  |  |  |  |  | 0.62 |  |  |  |  |
| 32 |  |  |  |  |  |  | 0.73 |  |  |  |  |
| 42 |  |  |  |  |  |  | 0.78 |  |  |  |  |
| 45 |  |  |  |  |  |  |  | 0.63 |  |  |  |
| 8 |  |  |  |  |  |  |  | 0.75 |  |  |  |
| 39 |  |  |  |  |  |  |  | 0.75 |  |  |  |
| 14 |  |  |  |  |  |  |  |  | 0.65 |  |  |
| 17 |  |  |  |  |  |  |  |  | 0.69 |  |  |
| 2 |  |  |  |  |  |  |  |  | 0.81 |  |  |
| 34 |  |  |  |  |  |  |  |  |  | 0.78 |  |
| 26 |  |  |  |  |  |  |  |  |  | 0.79 |  |
| 3 |  |  |  |  |  |  |  |  |  |  | 0.52 |
| 28 |  |  |  |  |  |  |  |  |  |  | 0.57 |
| 24 |  |  |  |  |  |  |  |  |  |  | 0.77 |

*Notes*: Factor Descriptions. 1: Inner Knowing; 2: Knowing Yourself; 3: Embodied Sensations; 4: Inner Voice, Apparent Communication with Non-Physical Beings; 5: Inner Knowing Through Touch; 6: Visualizing to Access or Affect; 7: Healing; 8: Physical Sensations from Other People; 9: Knowing the Future; 10: Knowing Through Dreams; and 11: Knowing Other’s Minds.

**Table 3. Eigenvalues for 46-item inventory**

|  | eigenvalues |
| --- | --- |
| 1 | 14.15 |
| 2 | 2.87 |
| 3 | 2.31 |
| 4 | 1.84 |
| 5 | 1.27 |
| 6 | 1.21 |
| 7 | 0.99 |
| 8 | 0.96 |
| 9 | 0.88 |
| 10 | 0.75 |
| 11 | 0.74 |
| 12 | 0.49 |
| 13 | 0.41 |
| 14 | 0.31 |
| 15 | 0.22 |
| 16 | 0.18 |
| 17 | 0.13 |
| 18 | 0.11 |
| 19 | 0.11 |
| 20 | 0.10 |
| 21 | 0.08 |
| 22 | 0.07 |
| 23 | 0.04 |
| 24 | 0.03 |
| 25 | 0.01 |
| 26 | 0.00 |
| 27 | -0.02 |
| 28 | -0.02 |
| 29 | -0.03 |
| 30 | -0.04 |
| 31 | -0.05 |
| 32 | -0.06 |
| 33 | -0.07 |
| 34 | -0.08 |
| 35 | -0.09 |
| 36 | -0.11 |
| 37 | -0.11 |
| 38 | -0.12 |
| 39 | -0.14 |
| 40 | -0.15 |
| 41 | -0.16 |
| 42 | -0.18 |
| 43 | -0.18 |
| 44 | -0.22 |
| 45 | -0.23 |
| 46 | -0.25 |

## STUDY 2

### Measures

**Demographic Race question**

Acknowledging that we are all part of the human race, which ethnicity(ies) BEST DESCRIBE(S) your current and most recent background? Please check all that apply.

- Native American. A person having origins in the indigenous peoples of North, Central, and South America.
- Native Pacific Islander. A person having origins in the indigenous peoples of Hawaii, Guam, Samoa, Australia or New Zealand, or other Pacific Islands.
- Asian. A person having origins in the Far East, Southeast Asia, or the Indian subcontinent including, for example, Cambodia, China, India, Japan, Korea, Malaysia, Pakistan, the Philippine Islands, Thailand, and Vietnam.
- African. A person having origins in any of the black ethnic groups of Africa (not including the Northern African Middle Eastern countries of Egypt, Libya, Sudan, Tunisia, Algeria, and Morocco).
- Middle Eastern. A person having origins in the Middle East including North Africa (Egypt, Libya, Sudan, Tunisia, Algeria, and Morocco).
- Latinx or Hispanic. A person having origins in Cuba, Mexico, Puerto Rico, South or Central America, or other Spanish culture or origin.
- European. A person having origins in Europe, including Scandinavia.

**Construct Validity Measures**

The scales selected have already been assessed as valid and reliable and used in numerous peer-reviewed publications. Correlation matrices of the scores were evaluated for expected patterns of associations between measures of the same construct.

*Convergent validity* was measured by administering two validated survey instruments that evaluate similar concepts, the Noetic Experience and Belief Scale and the Anomalous Experiences Inventory.

- The Noetic Experience and Belief Scale (NEBS) is a 20-item scale that measures paranormal belief and experience (Wahbeh et al., 2020). The scale contains ten statements about the belief in intuition, non-local consciousness, extraterrestrials, precognition, life after death, contact with the dead, clairvoyance, psychokinesis, telepathy, and automatism, which the participant rates on a slider from Disagree Strongly (0) to Agree Strongly (100). For each item, participants also answer whether they have ever personally experienced the phenomenon on a slider scale from Never (0) to Always (100). The scale results in global scores for paranormal belief and paranormal experience by averaging the ten-items for each subscale (0-100). Item scores can also be used individually for scores on each specific category (0-100). The belief subscale Cronbach’s alpha = 0.90; Experience subscale Cronbach’s alpha of 0.93. The NEBS had high test-retest reliability for both the belief (*r =* 0.83*, p* <0.00005) and experience values (*r =* 0.77*, p* <0.00005). While this scale does address noetic experiences, the items do not evaluate the wide breadth of characteristic ways people access inner wisdom. Thus, the NEBS included in this study allows us to evaluate convergent validity because it queries a similar construct.
- The Anomalous Experiences Inventory (AEI) is a 70-item questionnaire that evaluates multiple subscales related to anomalous experiences (Gallagher et al., 1994). The anomalous/paranormal experience and anomalous/paranormal ability subscales were used for this study for a total for 45 items. Respondents answer True (1) or False (0) for each item and values are summed for each scale. The AEI experience and ability scores are similar in concept to what the NSI is attempting to evaluate unique characteristics of noetic experiences that are not fully explored through the AEI. The AEI is also longer than is feasible for people to complete in many settings.

*Divergent validity* was measured by administering five validated survey instruments that evaluated different concepts than noetic phenomena: personality, emotional competence, high sensitivity, and psychotic and dissociative symptoms.

- Personality – 10 items (Rammstedt, 2007)
- *Brief Five Inventory - 10 (BFI-10)* The BFI-10 is a ten-item scale asking the participant to describe their personality on a rating scale of disagree strongly to agree strongly based on ten statements. These correspond to the big five personality characteristics of extraversion, agreeableness, conscientiousness, neuroticism, and openness with two items per subscale. After reverse scoring one item per subscale, item scores are averaged for subscale values. Overall, results indicate that the BFI-10 scales retain significant reliability and validity levels compared to the longer scales.
- Emotional competence – 20 items (Mikolajczak et al., 2014)
- *The Short Profile of Emotional Competence (S-PEC)* The S-PEC is a 20-item scale that evaluates people’s intrapersonal and interpersonal emotional competence with the following subscales for the two categories (Identification, Comprehension, Expression, Regulation, and Utilisation) with Cronbach alphas from 0.57 to 0.68. Total intrapersonal and interpersonal scores have alphas of 0.93 and 0.85, and a combined global score has an alpha of 0.85). Sample items include “When I am touched by something, I immediately know what I feel.”
- Highly sensitive person – 6-items (Aron & Aron, 1997)
- *Highly Sensitive Person Scale (HSPS)* is a 6-item self-report questionnaire that evaluates people’s sensitivity to the environment and emotions. The unidimensional scale has alphas of 0.65–0.85 across numerous samples and high test–retest reliability. Sample items include “Are you easily overwhelmed by things like bright lights, strong smells, coarse fabrics or sirens close by?” and “Do other people’s moods affect you?” Respondents reply on a 7-item Likert scale with 1 anchored at Not at All and 7 anchored at Extremely. Items are averaged for a total score.
- Psychotic symptoms 15 items (Capra et al., 2013)
- *The Community Assessment of Psychic Experiences- Positive Scale (CAPE-P15)* is a self-screening questionnaire to address subclinical positive psychotic symptoms in community contexts. It is valid, reliable, has the same three-factor structure as the lifetime version consisting of persecutory ideation, bizarre experiences, and perceptual abnormalities, and is highly predictive of generalized distress (*r* = .52). Each item is rated using a 4-point scale with the anchors, 0 (never), 1 (sometimes), 2 (often) and 3 (nearly always). The total score on the CAPE-P15 can range from 0 to 45. (Capra et al., 2015).
- Dissociative symptoms – 8 items (N. Waller et al., 1996)
- *Dissociation Experiences Scale Taxon (DES-T)* indexes pathological dissociation and has been shown to differentiate between psychiatric presentations that contain dissociative symptoms and those that do not(N. Waller et al., 1996). The DES-T is an eight-item subscale of the full-scale DES(N. G. Waller & Ross, 1997), Cronbach ɑ of 0.75 and is significantly correlated to the larger DES scale (*r* = 0.79)(Zingrone & Alvarado, 2001). Respondents selected a percentage number (e.g., 0% to 100%) indicating the frequency that they experienced the dissociative symptom. Each item was then scored on a scale from 1 to 100, and the overall score was the mean of the eight items. The DES-T distinguishes pathological dissociation more accurately than does the full-scale DES, with a cutoff score of 20 capturing nearly 90% of cases of pathological dissociation.

**11-Factor Model CFA Formulas for 46-item NSI**

“Q” indicates question and “R” indicates that the question was reverse coded.

F1 =~ Q4 + Q25 + Q27 + Q12 + Q13 + Q5 + Q11 + Q15 + Q23 + Q35

F2 =~ Q10 + Q18 + Q19 + Q43

F3 =~ Q1 + Q7 + Q29 + Q30 + Q40 + Q41

F4 =~ Q16 + Q21 + Q22 + Q36 + Q44R

F5 =~ Q31 + Q38 + Q46R

F6 =~ Q6R + Q20 + Q33 + Q37

F7 =~ Q9 + Q32 + Q42R

F8 =~ Q8 + Q39 + Q45

F9 =~ Q2 + Q14 + Q17

F10 =~ Q26R + Q34

F11 =~ Q3 + Q24 + Q28

**Table 3.** 11-Factor CFA fitted model using the maximum likelihood (ML) with bootstrap.

|  | Model | | | |
| --- | --- | --- | --- | --- |
|  | **Estimate** | **Std. Err.** | **z** | **p** |
|  | Factor Loadings | | | |
| F1 |  |  |  |  |
| Q4 | 13.70 | 1.13 | 12.09 | .000 |
| Q25 | 15.71 | 1.08 | 14.50 | .000 |
| Q27 | 12.33 | 0.84 | 14.62 | .000 |
| Q12 | 13.63 | 0.91 | 14.96 | .000 |
| Q13 | 13.00 | 0.99 | 13.07 | .000 |
| Q5 | 10.44 | 0.85 | 12.35 | .000 |
| Q11 | 10.85 | 0.83 | 13.13 | .000 |
| Q15 | 20.42 | 0.91 | 22.52 | .000 |
| Q23 | 22.52 | 0.97 | 23.23 | .000 |
| Q35 | 19.00 | 1.02 | 18.54 | .000 |
| F2 |  |  |  |  |
| Q10 | 13.52 | 0.99 | 13.67 | .000 |
| Q18 | 17.57 | 0.87 | 20.29 | .000 |
| Q19 | 11.58 | 0.70 | 16.54 | .000 |
| Q43 | 13.08 | 0.98 | 13.37 | .000 |
| F3 |  |  |  |  |
| Q1 | 17.51 | 1.09 | 16.09 | .000 |
| Q7 | 7.27 | 1.04 | 7.01 | .000 |
| Q29 | 18.59 | 1.08 | 17.15 | .000 |
| Q30 | 18.77 | 1.14 | 16.46 | .000 |
| Q40 | 23.66 | 1.06 | 22.28 | .000 |
| Q41 | 21.49 | 1.12 | 19.14 | .000 |
| F4 |  |  |  |  |
| Q16 | 23.77 | 1.13 | 21.03 | .000 |
| Q21 | 15.92 | 1.08 | 14.72 | .000 |
| Q22 | 26.50 | 1.00 | 26.39 | .000 |
| Q36 | 17.92 | 1.12 | 16.04 | .000 |
| Q44R | 22.93 | 1.22 | 18.83 | .000 |
| F5 |  |  |  |  |
| Q31 | 28.80 | 0.83 | 34.66 | .000 |
| Q38 | 29.76 | 0.76 | 39.27 | .000 |
| Q46R | 29.94 | 0.81 | 36.76 | .000 |
| F6 |  |  |  |  |
| Q6R | 22.24 | 1.17 | 19.02 | .000 |
| Q20 | 18.36 | 1.18 | 15.54 | .000 |
| Q33 | 25.73 | 1.00 | 25.82 | .000 |
| Q37 | 20.62 | 1.10 | 18.79 | .000 |
| F7 |  |  |  |  |
| Q9 | 22.92 | 1.01 | 22.72 | .000 |
| Q32 | 29.36 | 0.84 | 35.11 | .000 |
| Q42R | 30.87 | 0.91 | 34.03 | .000 |
| F8 |  |  |  |  |
| Q8 | 25.58 | 1.00 | 25.66 | .000 |
| Q39 | 29.59 | 0.91 | 32.69 | .000 |
| Q45 | 26.93 | 0.90 | 30.08 | .000 |
| F9 |  |  |  |  |
| Q2 | 20.88 | 1.04 | 20.05 | .000 |
| Q14 | 18.80 | 0.97 | 19.35 | .000 |
| Q17 | 20.18 | 1.06 | 19.03 | .000 |
| F10 |  |  |  |  |
| Q26R | 23.94 | 1.21 | 19.86 | .000 |
| Q34 | 27.24 | 1.19 | 22.94 | .000 |
| F11 |  |  |  |  |
| Q3 | 19.05 | 1.14 | 16.69 | .000 |
| Q24 | 24.35 | 1.00 | 24.25 | .000 |
| Q28 | 21.06 | 1.11 | 18.92 | .000 |
|  | Residual Variances | | | |
| Q4 | 258.33 | 32.37 | 7.98 | .000 |
| Q25 | 337.02 | 26.20 | 12.86 | .000 |
| Q27 | 186.27 | 17.39 | 10.71 | .000 |
| Q12 | 277.68 | 30.93 | 8.98 | .000 |
| Q13 | 311.17 | 30.50 | 10.20 | .000 |
| Q5 | 172.96 | 19.29 | 8.97 | .000 |
| Q11 | 222.04 | 21.86 | 10.16 | .000 |
| Q15 | 425.28 | 32.75 | 12.99 | .000 |
| Q23 | 271.63 | 22.76 | 11.93 | .000 |
| Q35 | 302.52 | 27.42 | 11.03 | .000 |
| Q10 | 309.21 | 31.26 | 9.89 | .000 |
| Q18 | 159.30 | 21.76 | 7.32 | .000 |
| Q19 | 121.75 | 13.10 | 9.29 | .000 |
| Q43 | 367.12 | 33.92 | 10.82 | .000 |
| Q1 | 606.21 | 37.53 | 16.15 | .000 |
| Q7 | 361.03 | 42.97 | 8.40 | .000 |
| Q29 | 447.27 | 31.19 | 14.34 | .000 |
| Q30 | 681.17 | 40.94 | 16.64 | .000 |
| Q40 | 503.26 | 44.41 | 11.33 | .000 |
| Q41 | 571.73 | 44.89 | 12.74 | .000 |
| Q16 | 720.73 | 51.01 | 14.13 | .000 |
| Q21 | 474.86 | 43.48 | 10.92 | .000 |
| Q22 | 507.62 | 45.96 | 11.04 | .000 |
| Q36 | 493.21 | 41.26 | 11.96 | .000 |
| Q44R | 512.35 | 44.13 | 11.61 | .000 |
| Q31 | 238.58 | 30.05 | 7.94 | .000 |
| Q38 | 147.59 | 21.85 | 6.76 | .000 |
| Q46R | 311.63 | 35.40 | 8.80 | .000 |
| Q6R | 653.67 | 52.38 | 12.48 | .000 |
| Q20 | 376.09 | 34.18 | 11.00 | .000 |
| Q33 | 174.63 | 32.23 | 5.42 | .000 |
| Q37 | 280.28 | 26.51 | 10.57 | .000 |
| Q9 | 284.64 | 27.33 | 10.41 | .000 |
| Q32 | 209.26 | 29.42 | 7.11 | .000 |
| Q42R | 257.41 | 37.32 | 6.90 | .000 |
| Q8 | 287.53 | 29.07 | 9.89 | .000 |
| Q39 | 155.97 | 23.98 | 6.50 | .000 |
| Q45 | 350.19 | 33.47 | 10.46 | .000 |
| Q2 | 174.06 | 24.59 | 7.08 | .000 |
| Q14 | 137.73 | 18.66 | 7.38 | .000 |
| Q17 | 195.26 | 27.96 | 6.98 | .000 |
| Q26R | 186.50 | 39.04 | 4.78 | .000 |
| Q34 | 30.36 | 46.77 | 0.65 | .516 |
| Q3 | 386.93 | 33.78 | 11.46 | .000 |
| Q24 | 258.68 | 34.07 | 7.59 | .000 |
| Q28 | 368.60 | 38.04 | 9.69 | .000 |
|  | Latent Variances | | | |
| F1 | 1.00^+^ |  |  |  |
| F2 | 1.00^+^ |  |  |  |
| F3 | 1.00^+^ |  |  |  |
| F4 | 1.00^+^ |  |  |  |
| F5 | 1.00^+^ |  |  |  |
| F6 | 1.00^+^ |  |  |  |
| F7 | 1.00^+^ |  |  |  |
| F8 | 1.00^+^ |  |  |  |
| F9 | 1.00^+^ |  |  |  |
| F10 | 1.00^+^ |  |  |  |
| F11 | 1.00^+^ |  |  |  |
|  | Latent Covariances | | | |
| F1 w/F2 | 0.42 | 0.05 | 8.42 | .000 |
| F1 w/F3 | 0.56 | 0.04 | 14.52 | .000 |
| F1 w/F4 | 0.47 | 0.04 | 11.39 | .000 |
| F1 w/F5 | 0.45 | 0.04 | 12.46 | .000 |
| F1 w/F6 | 0.36 | 0.04 | 8.29 | .000 |
| F1 w/F7 | 0.43 | 0.04 | 10.12 | .000 |
| F1 w/F8 | 0.56 | 0.04 | 15.29 | .000 |
| F1 w/F9 | 0.58 | 0.04 | 15.04 | .000 |
| F1 w/F10 | 0.28 | 0.04 | 6.28 | .000 |
| F1 w/F11 | 0.65 | 0.04 | 16.11 | .000 |
| F2 w/F3 | 0.27 | 0.05 | 5.40 | .000 |
| F2 w/F4 | 0.55 | 0.04 | 13.61 | .000 |
| F2 w/F5 | 0.27 | 0.04 | 6.09 | .000 |
| F2 w/F6 | 0.52 | 0.04 | 13.26 | .000 |
| F2 w/F7 | 0.45 | 0.04 | 10.97 | .000 |
| F2 w/F8 | 0.26 | 0.05 | 5.80 | .000 |
| F2 w/F9 | 0.28 | 0.05 | 5.58 | .000 |
| F2 w/F10 | 0.27 | 0.05 | 5.46 | .000 |
| F2 w/F11 | 0.36 | 0.05 | 7.20 | .000 |
| F3 w/F4 | 0.53 | 0.04 | 13.57 | .000 |
| F3 w/F5 | 0.57 | 0.04 | 14.01 | .000 |
| F3 w/F6 | 0.31 | 0.04 | 7.33 | .000 |
| F3 w/F7 | 0.41 | 0.04 | 10.10 | .000 |
| F3 w/F8 | 0.61 | 0.04 | 16.23 | .000 |
| F3 w/F9 | 0.36 | 0.04 | 8.27 | .000 |
| F3 w/F10 | 0.24 | 0.04 | 5.51 | .000 |
| F3 w/F11 | 0.53 | 0.04 | 12.96 | .000 |
| F4 w/F5 | 0.49 | 0.04 | 12.21 | .000 |
| F4 w/F6 | 0.58 | 0.04 | 15.06 | .000 |
| F4 w/F7 | 0.47 | 0.04 | 11.44 | .000 |
| F4 w/F8 | 0.41 | 0.04 | 9.26 | .000 |
| F4 w/F9 | 0.36 | 0.05 | 7.39 | .000 |
| F4 w/F10 | 0.35 | 0.05 | 7.66 | .000 |
| F4 w/F11 | 0.58 | 0.04 | 13.78 | .000 |
| F5 w/F6 | 0.32 | 0.04 | 8.09 | .000 |
| F5 w/F7 | 0.46 | 0.04 | 11.94 | .000 |
| F5 w/F8 | 0.51 | 0.04 | 13.89 | .000 |
| F5 w/F9 | 0.35 | 0.04 | 8.69 | .000 |
| F5 w/F10 | 0.18 | 0.04 | 4.01 | .000 |
| F5 w/F11 | 0.47 | 0.04 | 11.81 | .000 |
| F6 w/F7 | 0.48 | 0.04 | 12.16 | .000 |
| F6 w/F8 | 0.33 | 0.04 | 7.94 | .000 |
| F6 w/F9 | 0.26 | 0.05 | 5.05 | .000 |
| F6 w/F10 | 0.32 | 0.05 | 7.09 | .000 |
| F6 w/F11 | 0.43 | 0.05 | 8.94 | .000 |
| F7 w/F8 | 0.54 | 0.04 | 14.81 | .000 |
| F7 w/F9 | 0.26 | 0.05 | 5.52 | .000 |
| F7 w/F10 | 0.19 | 0.05 | 4.08 | .000 |
| F7 w/F11 | 0.46 | 0.04 | 10.70 | .000 |
| F8 w/F9 | 0.34 | 0.04 | 7.75 | .000 |
| F8 w/F10 | 0.21 | 0.04 | 4.77 | .000 |
| F8 w/F11 | 0.53 | 0.04 | 13.06 | .000 |
| F9 w/F10 | 0.33 | 0.05 | 7.14 | .000 |
| F9 w/F11 | 0.52 | 0.04 | 12.02 | .000 |
| F10 w/F11 | 0.26 | 0.04 | 5.83 | .000 |
|  | Fit Indices | | | |
| NPAR | 147.00 |  |  |  |
| FMIN | 3.13 |  |  |  |
| χ^2^ | 4188.95 |  |  |  |
| DF | 934.00 |  |  |  |
| PVALUE | 0.00 |  |  |  |
| BASELINE.CHISQ | 19212.60 |  |  |  |
| BASELINE.DF | 1035.00 |  |  |  |
| BASELINE.PVALUE | 0.00 |  |  |  |
| CFI | 0.82 |  |  |  |
| TLI | 0.80 |  |  |  |
| NNFI | 0.80 |  |  |  |
| RFI | 0.76 |  |  |  |
| NFI | 0.78 |  |  |  |
| PNFI | 0.71 |  |  |  |
| IFI | 0.82 |  |  |  |
| RNI | 0.82 |  |  |  |
| LOGL | -137450.02 |  |  |  |
| UNRESTRICTED.LOGL | -135355.55 |  |  |  |
| AIC | 275194.04 |  |  |  |
| BIC | 275856.39 |  |  |  |
| NTOTAL | 669.00 |  |  |  |
| BIC2 | 275389.66 |  |  |  |
| RMSEA | 0.07 |  |  |  |
| RMSEA.CI.LOWER | 0.07 |  |  |  |
| RMSEA.CI.UPPER | 0.07 |  |  |  |
| RMSEA.PVALUE | 0.00 |  |  |  |
| RMR | 46.98 |  |  |  |
| RMR_NOMEAN | 46.98 |  |  |  |
| SRMR | 0.07 |  |  |  |
| SRMR_BENTLER | 0.07 |  |  |  |
| SRMR_BENTLER_NOMEAN | 0.07 |  |  |  |
| CRMR | 0.07 |  |  |  |
| CRMR_NOMEAN | 0.07 |  |  |  |
| SRMR_MPLUS | 0.07 |  |  |  |
| SRMR_MPLUS_NOMEAN | 0.07 |  |  |  |
| CN_05 | 161.70 |  |  |  |
| CN_01 | 166.69 |  |  |  |
| GFI | 0.78 |  |  |  |
| AGFI | 0.74 |  |  |  |
| PGFI | 0.67 |  |  |  |
| MFI | 0.09 |  |  |  |
| ECVI | 6.70 |  |  |  |
| Scaled χ^2^ | 4188.95(934) |  |  | .000 |
| ^+^Fixed parameter | | | | |

**Table 4. Statistical output of EFA with larger dataset (44-item subset of 175-item NSI + 44-item NSI)**

|  | SS loadings | Proportion Var | Cumulative Var | Proportion Explained | Cumulative Proportion |
| --- | --- | --- | --- | --- | --- |
| 1 | 5.03 | 0.11 | 0.11 | 0.18 | 0.18 |
| 10 | 2.7 | 0.06 | 0.18 | 0.1 | 0.28 |
| 8 | 2.52 | 0.06 | 0.23 | 0.09 | 0.37 |
| 3 | 2.38 | 0.05 | 0.29 | 0.09 | 0.46 |
| 2 | 2.18 | 0.05 | 0.34 | 0.08 | 0.54 |
| 9 | 2.18 | 0.05 | 0.39 | 0.08 | 0.62 |
| 5 | 2.01 | 0.05 | 0.43 | 0.07 | 0.69 |
| 7 | 1.79 | 0.04 | 0.47 | 0.07 | 0.76 |
| 11 | 1.77 | 0.04 | 0.51 | 0.06 | 0.82 |
| 12 | 1.7 | 0.04 | 0.55 | 0.06 | 0.89 |
| 4 | 1.64 | 0.04 | 0.59 | 0.06 | 0.95 |
| 6 | 1.46 | 0.03 | 0.62 | 0.05 | 1 |

Tucker Lewis Index of factoring reliability = 0.943

RMSEA index = 0.038 and the 90 % confidence intervals are 0.036 - 0.041

**12-Factor Model Formulas for 44-item NSI**

12 factors labeled with “F” and the factor number according to the following formula:

F1 =~ Q4 + Q5 + Q10 + Q11 + Q12 + Q14 + Q22 + Q24 + Q26 + Q34

F2 =~ Q8 + Q31 + Q41R

F3 =~ Q30 + Q37 + Q44R

F4 =~ Q25R + Q33

F5 =~ Q7 + Q38 + Q43

F6 =~ Q20 + Q35

F7 =~ Q9 + Q17 + Q18

F8 =~ Q6R + Q19 + Q32 + Q36

F9 =~ Q2 + Q13 + Q16

F10 =~ Q1 + Q28 + Q29 + Q39 + Q40

F11 =~ Q3 + Q23 + Q27

F12 =~ Q15 + Q21 + Q42R

“Q” indicates question and “R” indicates that the question was reverse coded.

**Table 5. Factor Loadings for each item of the 44-item NSI.**

| **OLD 46 NSI Q #** | **NEW 44 NSI Q #** | **1** (1) | **2** (3) | **3** (6) | **4** (5) | **5** (7) | **6** (9) | **7** (8) | **8** (2) | **9** (11) | **10** (4) | **11** (10) | **12** (4) |
| --- | --- | --- | --- | --- | --- | --- | --- | --- | --- | --- | --- | --- | --- |
| 4 | 4 | 0.67 |  |  |  |  |  |  |  |  |  |  |  |
| 25 | 24 | 0.50 |  |  |  |  |  |  |  |  |  |  |  |
| 27 | 26 | 0.67 |  |  |  |  |  |  |  |  |  |  |  |
| 12 | 11 | 0.66 |  |  |  |  |  |  |  |  |  |  |  |
| 13 | 12 | 0.59 |  |  |  |  |  |  |  |  |  |  |  |
| 5 | 5 | 0.68 |  |  |  |  |  |  |  |  |  |  |  |
| 11 | 10 | 0.63 |  |  |  |  |  |  |  |  |  |  |  |
| 15 | 14 | 0.52 |  |  |  |  |  |  |  |  |  |  |  |
| 23 | 22 | 0.68 |  |  |  |  |  |  |  |  |  |  |  |
| 35 | 34 | 0.56 |  |  |  |  |  |  |  |  |  |  |  |
| 1 | **1** |  | 0.59 |  |  |  |  |  |  |  |  |  |  |
| 29 | **28** |  | 0.63 |  |  |  |  |  |  |  |  |  |  |
| 30 | **29** |  | 0.54 |  |  |  |  |  |  |  |  |  |  |
| 40 | **39** |  | 0.57 |  |  |  |  |  |  |  |  |  |  |
| 41 | **40** |  | 0.63 |  |  |  |  |  |  |  |  |  |  |
| 6R | 6R |  |  | 0.65 |  |  |  |  |  |  |  |  |  |
| 20 | 19 |  |  | 0.57 |  |  |  |  |  |  |  |  |  |
| 33 | 32 |  |  | 0.88 |  |  |  |  |  |  |  |  |  |
| 37 | 36 |  |  | 0.61 |  |  |  |  |  |  |  |  |  |
| 31 | 30 |  |  |  | 0.83 |  |  |  |  |  |  |  |  |
| 38 | 37 |  |  |  | 0.78 |  |  |  |  |  |  |  |  |
| 46R | 44R |  |  |  | 0.70 |  |  |  |  |  |  |  |  |
| 9 | 8 |  |  |  |  | 0.66 |  |  |  |  |  |  |  |
| 32 | 31 |  |  |  |  | 0.77 |  |  |  |  |  |  |  |
| 42R | 41R |  |  |  |  | 0.82 |  |  |  |  |  |  |  |
| 2 | 2 |  |  |  |  |  | 0.78 |  |  |  |  |  |  |
| 14 | 13 |  |  |  |  |  | 0.72 |  |  |  |  |  |  |
| 17 | 16 |  |  |  |  |  | 0.74 |  |  |  |  |  |  |
| 8 | 7 |  |  |  |  |  |  | 0.72 |  |  |  |  |  |
| 39 | 38 |  |  |  |  |  |  | 0.77 |  |  |  |  |  |
| 45 | 43 |  |  |  |  |  |  | 0.63 |  |  |  |  |  |
| 10 | 9 |  |  |  |  |  |  |  | 0.66 |  |  |  |  |
| 18 | 17 |  |  |  |  |  |  |  | 0.71 |  |  |  |  |
| 19 | 18 |  |  |  |  |  |  |  | 0.59 |  |  |  |  |
| 3 | 3 |  |  |  |  |  |  |  |  | 0.53 |  |  |  |
| 24 | 23 |  |  |  |  |  |  |  |  | 0.75 |  |  |  |
| 28 | 27 |  |  |  |  |  |  |  |  | 0.56 |  |  |  |
| 16 | 15 |  |  |  |  |  |  |  |  |  | 0.59 |  |  |
| 22 | 21 |  |  |  |  |  |  |  |  |  | 0.59 |  |  |
| 44R | 42R |  |  |  |  |  |  |  |  |  | 0.70 |  |  |
| 26R | 25R |  |  |  |  |  |  |  |  |  |  | 0.93 |  |
| 34 | 33 |  |  |  |  |  |  |  |  |  |  | 0.77 |  |
| 21 | 20 |  |  |  |  |  |  |  |  |  |  |  | 0.76 |
| 36 | 35 |  |  |  |  |  |  |  |  |  |  |  | 0.78 |

*Notes*: Column 1 is the 46-item NSI question number. Column 2 is the 44-item question number. Factors 1 through 12 with the factor loadings for each factor are listed. Below each factor number for the 12-factor model is the corresponding factor in the 11-factor model. Factor Descriptions. 1: Inner Knowing; 2: Embodied Sensations; 3: Visualizing to Access or Affect; 4: Inner Knowing Through Touch; 5: Healing; 6: Knowing the Future; 7: Physical Sensations from Other People; 8: Knowing Yourself; 9: Knowing Other’s Minds; 10: Apparent Communication with Non-Physical Beings; 11: Knowing Through Dreams; and 12: Inner Voice.

**Table 6. Eigenvalues for 44-tiem NSI**

|  | eigenvalues |
| --- | --- |
| 1 | 12.94 |
| 2 | 2.60 |
| 3 | 2.33 |
| 4 | 1.82 |
| 5 | 1.32 |
| 6 | 1.25 |
| 7 | 1.08 |
| 8 | 1.00 |
| 9 | 0.91 |
| 10 | 0.80 |
| 11 | 0.74 |
| 12 | 0.57 |
| 13 | 0.33 |
| 14 | 0.22 |
| 15 | 0.21 |
| 16 | 0.12 |
| 17 | 0.11 |
| 18 | 0.08 |
| 19 | 0.07 |
| 20 | 0.06 |
| 21 | 0.05 |
| 22 | 0.04 |
| 23 | 0.04 |
| 24 | 0.02 |
| 25 | 0.01 |
| 26 | 0.01 |
| 27 | 0.00 |
| 28 | 0.00 |
| 29 | -0.02 |
| 30 | -0.03 |
| 31 | -0.04 |
| 32 | -0.04 |
| 33 | -0.05 |
| 34 | -0.06 |
| 35 | -0.07 |
| 36 | -0.07 |
| 37 | -0.08 |
| 38 | -0.09 |
| 39 | -0.10 |
| 40 | -0.12 |
| 41 | -0.13 |
| 42 | -0.14 |
| 43 | -0.16 |
| 44 | -0.17 |

## STUDY 3

Data Quality Control

There were five reverse coded item pairs. The values for these pairs were subtracted from each other to create difference variables. A preliminary valid/not valid categorization was assigned by applying *k*-means and hierarchical clustering to only the difference variables. This assignment was then modified manually using visual assessment, with ‘maybe valid’ as a possible category. Then, survey responses known to be ‘not valid’ were added to this data set. ‘Maybe valid’ responses were withheld (prediction data set), while ‘valid’ and ‘not valid’ responses were used to train and test machine learning models (model building dataset). Specifically, 75% of the data from the model building dataset was randomly allocated to a training dataset, while the remaining 25% was allocated to a test dataset.

Random forests, support vector machines (linear, polynomial, radial, and sigmoid kernels), neural networks, and quadratic discriminant analysis models were trained using the training data, and their performance was evaluated using the test dataset. The best performing model was used to generate predictions for the prediction data set, and in this way, all ‘Maybe valid’ assignments were replaced with the model predicted ‘valid’ or ‘no valid’ assignment. The model building dataset and updated prediction dataset were then re-joined for use in factor analysis.

**Table 6.** CFA fitted model using diagonally weighted least squares.

|  | Model | | | |
| --- | --- | --- | --- | --- |
|  | Estimate | Std. Err. | z | p |
|  | Factor Loadings | | | |
| F1 |  |  |  |  |
| Q4 | 13.13 | 0.30 | 44.25 | .000 |
| Q5 | 10.33 | 0.24 | 42.27 | .000 |
| Q10 | 13.28 | 0.29 | 46.00 | .000 |
| Q11 | 10.78 | 0.28 | 39.03 | .000 |
| Q12 | 13.33 | 0.29 | 46.07 | .000 |
| Q14 | 22.51 | 0.34 | 65.45 | .000 |
| Q22 | 23.72 | 0.36 | 66.14 | .000 |
| Q24 | 25.79 | 0.37 | 70.00 | .000 |
| Q26 | 15.11 | 0.31 | 49.33 | .000 |
| Q34 | 23.73 | 0.36 | 65.66 | .000 |
| F2 |  |  |  |  |
| Q8 | 23.34 | 0.53 | 43.84 | .000 |
| Q31 | 26.09 | 0.59 | 44.42 | .000 |
| Q41R | 18.79 | 0.48 | 38.75 | .000 |
| F3 |  |  |  |  |
| Q30 | 26.27 | 0.51 | 51.92 | .000 |
| Q37 | 27.87 | 0.53 | 52.28 | .000 |
| Q44R | 22.74 | 0.47 | 48.03 | .000 |
| F4 |  |  |  |  |
| Q25R | 9.38 | 0.69 | 13.66 | .000 |
| Q33 | 30.67 | 2.25 | 13.66 | .000 |
| F5 |  |  |  |  |
| Q7 | 21.92 | 0.47 | 46.91 | .000 |
| Q38 | 26.45 | 0.53 | 50.00 | .000 |
| Q43 | 26.59 | 0.53 | 50.06 | .000 |
| F6 |  |  |  |  |
| Q20 | 25.34 | 0.67 | 38.09 | .000 |
| Q35 | 27.99 | 0.73 | 38.09 | .000 |
| F7 |  |  |  |  |
| Q9 | 17.39 | 0.44 | 39.39 | .000 |
| Q17 | 24.65 | 0.58 | 42.81 | .000 |
| Q18 | 21.35 | 0.51 | 41.59 | .000 |
| F8 |  |  |  |  |
| Q6R | 6.15 | 0.32 | 19.18 | .000 |
| Q19 | 22.59 | 0.44 | 50.99 | .000 |
| Q32 | 26.09 | 0.48 | 54.11 | .000 |
| Q36 | 26.17 | 0.48 | 54.06 | .000 |
| F9 |  |  |  |  |
| Q2 | 19.68 | 0.52 | 37.86 | .000 |
| Q13 | 19.49 | 0.51 | 38.45 | .000 |
| Q16 | 22.88 | 0.58 | 39.19 | .000 |
| F10 |  |  |  |  |
| Q1 | 15.51 | 0.34 | 46.08 | .000 |
| Q28 | 21.37 | 0.38 | 55.78 | .000 |
| Q29 | 19.20 | 0.37 | 51.62 | .000 |
| Q39 | 23.30 | 0.40 | 57.92 | .000 |
| Q40 | 23.86 | 0.41 | 58.20 | .000 |
| F11 |  |  |  |  |
| Q3 | 14.60 | 0.39 | 37.89 | .000 |
| Q23 | 25.72 | 0.56 | 45.86 | .000 |
| Q27 | 26.18 | 0.57 | 45.81 | .000 |
| F12 |  |  |  |  |
| Q15 | 24.32 | 0.51 | 47.37 | .000 |
| Q21 | 27.89 | 0.56 | 49.57 | .000 |
| Q42R | 23.95 | 0.52 | 46.14 | .000 |
|  | Residual Variances | | | |
| Q4 | 449.74 | 35.46 | 12.68 | .000 |
| Q5 | 333.07 | 28.06 | 11.87 | .000 |
| Q10 | 417.89 | 31.76 | 13.16 | .000 |
| Q11 | 448.23 | 33.33 | 13.45 | .000 |
| Q12 | 440.31 | 34.08 | 12.92 | .000 |
| Q14 | 396.58 | 30.58 | 12.97 | .000 |
| Q22 | 365.88 | 36.23 | 10.10 | .000 |
| Q24 | 288.19 | 37.70 | 7.64 | .000 |
| Q26 | 428.60 | 36.51 | 11.74 | .000 |
| Q34 | 388.59 | 37.23 | 10.44 | .000 |
| Q8 | 436.25 | 36.99 | 11.79 | .000 |
| Q31 | 383.08 | 45.69 | 8.38 | .000 |
| Q41R | 893.70 | 39.77 | 22.47 | .000 |
| Q30 | 334.91 | 40.90 | 8.19 | .000 |
| Q37 | 270.07 | 46.03 | 5.87 | .000 |
| Q44R | 737.49 | 39.28 | 18.77 | .000 |
| Q25R | 968.67 | 35.72 | 27.12 | .000 |
| Q33 | 192.25 | 141.92 | 1.35 | .176 |
| Q7 | 599.71 | 34.74 | 17.26 | .000 |
| Q38 | 481.55 | 40.03 | 12.03 | .000 |
| Q43 | 419.91 | 40.32 | 10.41 | .000 |
| Q20 | 405.74 | 45.19 | 8.98 | .000 |
| Q35 | 335.12 | 50.52 | 6.63 | .000 |
| Q9 | 501.14 | 33.44 | 14.99 | .000 |
| Q17 | 299.35 | 40.78 | 7.34 | .000 |
| Q18 | 363.88 | 39.18 | 9.29 | .000 |
| Q6R | 847.61 | 27.09 | 31.28 | .000 |
| Q19 | 523.01 | 34.68 | 15.08 | .000 |
| Q32 | 374.74 | 38.34 | 9.77 | .000 |
| Q36 | 364.86 | 37.31 | 9.78 | .000 |
| Q2 | 478.89 | 37.48 | 12.78 | .000 |
| Q13 | 310.63 | 38.97 | 7.97 | .000 |
| Q16 | 368.41 | 43.71 | 8.43 | .000 |
| Q1 | 668.09 | 28.89 | 23.13 | .000 |
| Q28 | 511.70 | 35.85 | 14.27 | .000 |
| Q29 | 635.23 | 31.73 | 20.02 | .000 |
| Q39 | 483.16 | 36.32 | 13.30 | .000 |
| Q40 | 492.87 | 34.02 | 14.49 | .000 |
| Q3 | 571.25 | 29.61 | 19.29 | .000 |
| Q23 | 360.68 | 41.47 | 8.70 | .000 |
| Q27 | 378.76 | 42.51 | 8.91 | .000 |
| Q15 | 518.33 | 45.34 | 11.43 | .000 |
| Q21 | 283.23 | 45.23 | 6.26 | .000 |
| Q42R | 742.22 | 40.50 | 18.33 | .000 |
|  | Latent Variances | | | |
| F1 | 1.00^+^ |  |  |  |
| F2 | 1.00^+^ |  |  |  |
| F3 | 1.00^+^ |  |  |  |
| F4 | 1.00^+^ |  |  |  |
| F5 | 1.00^+^ |  |  |  |
| F6 | 1.00^+^ |  |  |  |
| F7 | 1.00^+^ |  |  |  |
| F8 | 1.00^+^ |  |  |  |
| F9 | 1.00^+^ |  |  |  |
| F10 | 1.00^+^ |  |  |  |
| F11 | 1.00^+^ |  |  |  |
| F12 | 1.00^+^ |  |  |  |
|  | Latent Covariances | | | |
| F1 w/F2 | 0.54 | 0.02 | 30.45 | .000 |
| F1 w/F3 | 0.56 | 0.02 | 35.97 | .000 |
| F1 w/F4 | 0.51 | 0.04 | 12.47 | .000 |
| F1 w/F5 | 0.66 | 0.02 | 37.42 | .000 |
| F1 w/F6 | 0.66 | 0.02 | 29.40 | .000 |
| F1 w/F7 | 0.75 | 0.02 | 34.45 | .000 |
| F1 w/F8 | 0.62 | 0.02 | 38.14 | .000 |
| F1 w/F9 | 0.76 | 0.02 | 33.57 | .000 |
| F1 w/F10 | 0.62 | 0.01 | 43.19 | .000 |
| F1 w/F11 | 0.60 | 0.02 | 32.90 | .000 |
| F1 w/F12 | 0.45 | 0.01 | 30.33 | .000 |
| F2 w/F3 | 0.80 | 0.03 | 27.80 | .000 |
| F2 w/F4 | 0.42 | 0.04 | 10.07 | .000 |
| F2 w/F5 | 0.73 | 0.03 | 26.06 | .000 |
| F2 w/F6 | 0.57 | 0.03 | 19.41 | .000 |
| F2 w/F7 | 0.63 | 0.03 | 22.49 | .000 |
| F2 w/F8 | 0.75 | 0.03 | 27.37 | .000 |
| F2 w/F9 | 0.45 | 0.03 | 17.49 | .000 |
| F2 w/F10 | 0.80 | 0.03 | 30.41 | .000 |
| F2 w/F11 | 0.70 | 0.03 | 23.83 | .000 |
| F2 w/F12 | 0.74 | 0.03 | 26.22 | .000 |
| F3 w/F4 | 0.44 | 0.04 | 10.74 | .000 |
| F3 w/F5 | 0.70 | 0.02 | 28.27 | .000 |
| F3 w/F6 | 0.60 | 0.03 | 21.84 | .000 |
| F3 w/F7 | 0.60 | 0.02 | 24.23 | .000 |
| F3 w/F8 | 0.77 | 0.02 | 30.86 | .000 |
| F3 w/F9 | 0.48 | 0.02 | 20.72 | .000 |
| F3 w/F10 | 0.87 | 0.02 | 35.65 | .000 |
| F3 w/F11 | 0.72 | 0.03 | 26.72 | .000 |
| F3 w/F12 | 0.76 | 0.03 | 29.27 | .000 |
| F4 w/F5 | 0.39 | 0.04 | 9.89 | .000 |
| F4 w/F6 | 0.52 | 0.05 | 10.51 | .000 |
| F4 w/F7 | 0.52 | 0.05 | 10.77 | .000 |
| F4 w/F8 | 0.51 | 0.05 | 11.29 | .000 |
| F4 w/F9 | 0.64 | 0.06 | 11.37 | .000 |
| F4 w/F10 | 0.49 | 0.04 | 11.58 | .000 |
| F4 w/F11 | 0.41 | 0.04 | 10.07 | .000 |
| F4 w/F12 | 0.45 | 0.04 | 10.85 | .000 |
| F5 w/F6 | 0.58 | 0.03 | 20.64 | .000 |
| F5 w/F7 | 0.63 | 0.03 | 23.64 | .000 |
| F5 w/F8 | 0.67 | 0.02 | 27.89 | .000 |
| F5 w/F9 | 0.53 | 0.03 | 20.95 | .000 |
| F5 w/F10 | 0.78 | 0.02 | 33.20 | .000 |
| F5 w/F11 | 0.69 | 0.03 | 25.60 | .000 |
| F5 w/F12 | 0.60 | 0.02 | 25.00 | .000 |
| F6 w/F7 | 0.69 | 0.03 | 21.13 | .000 |
| F6 w/F8 | 0.68 | 0.03 | 23.40 | .000 |
| F6 w/F9 | 0.59 | 0.03 | 19.04 | .000 |
| F6 w/F10 | 0.66 | 0.03 | 24.73 | .000 |
| F6 w/F11 | 0.71 | 0.03 | 22.63 | .000 |
| F6 w/F12 | 0.61 | 0.03 | 21.83 | .000 |
| F7 w/F8 | 0.73 | 0.03 | 26.87 | .000 |
| F7 w/F9 | 0.65 | 0.03 | 21.31 | .000 |
| F7 w/F10 | 0.73 | 0.03 | 29.08 | .000 |
| F7 w/F11 | 0.62 | 0.03 | 22.65 | .000 |
| F7 w/F12 | 0.60 | 0.03 | 23.68 | .000 |
| F8 w/F9 | 0.54 | 0.02 | 21.87 | .000 |
| F8 w/F10 | 0.80 | 0.02 | 35.05 | .000 |
| F8 w/F11 | 0.73 | 0.03 | 27.38 | .000 |
| F8 w/F12 | 0.70 | 0.02 | 28.70 | .000 |
| F9 w/F10 | 0.51 | 0.02 | 22.93 | .000 |
| F9 w/F11 | 0.50 | 0.03 | 19.10 | .000 |
| F9 w/F12 | 0.46 | 0.02 | 19.51 | .000 |
| F10 w/F11 | 0.72 | 0.02 | 29.31 | .000 |
| F10 w/F12 | 0.73 | 0.02 | 31.79 | .000 |
| F11 w/F12 | 0.73 | 0.03 | 26.52 | .000 |
|  | Fit Indices | | | |
| NPAR | 154.00 |  |  |  |
| FMIN | 0.89 |  |  |  |
| χ^2^ | 1800.39(836) |  |  | .000 |
| DF | 836.00 |  |  |  |
| PVALUE | 0.00 |  |  |  |
| BASELINE.CHISQ | 93435.21 |  |  |  |
| BASELINE.DF | 946.00 |  |  |  |
| BASELINE.PVALUE | 0.00 |  |  |  |
| CFI | 0.99 |  |  |  |
| TLI | 0.99 |  |  |  |
| NNFI | 0.99 |  |  |  |
| RFI | 0.98 |  |  |  |
| NFI | 0.98 |  |  |  |
| PNFI | 0.87 |  |  |  |
| IFI | 0.99 |  |  |  |
| RNI | 0.99 |  |  |  |
| RMSEA | 0.03 |  |  |  |
| RMSEA.CI.LOWER | 0.03 |  |  |  |
| RMSEA.CI.UPPER | 0.04 |  |  |  |
| RMSEA.PVALUE | 1.00 |  |  |  |
| RMR | 42.48 |  |  |  |
| RMR_NOMEAN | 42.48 |  |  |  |
| SRMR | 0.05 |  |  |  |
| SRMR_BENTLER | 0.05 |  |  |  |
| SRMR_BENTLER_NOMEAN | 0.05 |  |  |  |
| CRMR | 0.05 |  |  |  |
| CRMR_NOMEAN | 0.05 |  |  |  |
| SRMR_MPLUS | 0.05 |  |  |  |
| SRMR_MPLUS_NOMEAN | 0.05 |  |  |  |
| CN_05 | 508.85 |  |  |  |
| CN_01 | 525.51 |  |  |  |
| GFI | 0.99 |  |  |  |
| AGFI | 0.98 |  |  |  |
| PGFI | 0.83 |  |  |  |
| MFI | 0.62 |  |  |  |
| ECVI | 2.09 |  |  |  |
| ^+^Fixed parameter | | | | |

Final 44-item Noetic Signature Inventory

| **Question Number** | **Question Category** |
| --- | --- |
| 1 | Sensorial - Other Senses (From either external stimuli, NOT the person’s “inner voice” or “visions”*) -* Vestibular/Movement |
| 2 | Intuitive  - Premonition/Precognition  (Telling the future or knowing something will happen. May ALSO be coded with types of information coming from this way of knowing/Warnings) |
| 3 | Others - Thoughts from other people  (Knowing what someone was thinking about) |
| 4 | Others - Emotions/feelings from other people  (Has to be about someone else. i.e., “I knew they felt xyz”) |
| 5 | Intuitive - Intuition/"Just knowing"  (MUST be a “sense” of knowing/”a gut feeling” that is not embodied) |
| 6 | Intentional - Visualization |
| 7 | Others - Physical sensations from other people  (e.g. someone’s knee is hurting and they feel it) |
| 8 | Healing other |
| 9 | Self - Impact on Self from Others (People, spirits, environment, objects, etc. as impacting the self in some way) |
| 10 | Intuitive - Intuition/"Just knowing"  (MUST be a “sense” of knowing/”a gut feeling” that is not embodied) |
| 11 | Embodied - Accessing Body's Knowledge/“Just Feel It” (Any mention of the body itself as containing knowledge beyond the conscious self, MUST be referenced as the body primarily owning that knowledge) |
| 12 | Embodied - Physical Sensation - Solar plexus/gut sensation  (not intuitive but physical) |
| 13 | Intuitive  - Premonition/Precognition  (Telling the future or knowing something will happen. May ALSO be coded with types of information coming from this way of knowing/Warnings) |
| 14 | Intuitive - Intuition/"Just knowing"  (MUST be a “sense” of knowing/”a gut feeling” that is not embodied) |
| 15 | Direct - Communication with Others - Deceased People |
| 16 | Intuitive  - Premonition/Precognition  (Telling the future or knowing something will happen. May ALSO be coded with types of information coming from this way of knowing/Warnings) |
| 17 | Self - Sees Self in Certain Role  (As a result of the experience, the perception of what they do changes. Ie. “I realized I was a healer”)  ; Self  - Self-Awareness/Introspection/Reflection  (Trauma/Reflecting on the self, whether in self-improvement or understanding something about oneself, NOT as being in a certain role) |
| 18 | Impacts Decision Making  (Knowledge received informs how someone does something) |
| 19 | Intentional - Manifestation |
| 20 | Sensorial  - Inner Voice/Auditory  (MUST be from the person's “inner voice”) |
| 21 | Intentional - Channeling  (Can Also Be Automatic Writing/Speaking) |
| 22 | Direct-Communication with Others  - People |
| 23 | Relational - Telepathy/Mind to Mind Communication (Communication that is NOT verbal, but telepathic or in the mind. Must be actively communicating something, either one sided or both) |
| 24 | Embodied - Emotional |
| 25 | Intentional or Unintentional - Dreams/Sleep (not lucid dreaming) |
| 26 | Others - Emotions/feelings from other people  (Has to be about someone else. i.e., “I knew they felt xyz”) |
| 27 | Relational - Telepathy/Mind to Mind Communication (Communication that is NOT verbal, but telepathic or in the mind. Must be actively communicating something, either one sided or both) |
| 28 | Sensorial  - Other Senses (From either external stimuli, NOT the person’s “inner voice” or “visions”*)* - Taste |
| 29 | Embodied - Physical Sensation - Goosebumps |
| 30 | Senses - Direct Touch |
| 31 | Healing other |
| 32 | Intentional - Visualization |
| 33 | Intentional or Unintentional - Dreams/Sleep (not lucid dreaming) |
| 34 | Others - Information About Other People (not otherwise listed. i.e., “I knew they would do this, or they have an illness, etc.) |
| 35 | Sensorial  - Inner Voice/Auditory  (MUST be from the person's “inner voice”) |
| 36 | Intentional - Visualization |
| 37 | Senses - Direct Touch |
| 38 | Others - Physical sensations from other people  (e.g., someone’s knee is hurting and they feel it) |
| 39 | Sensorial - Other Senses (From either external stimuli, NOT the person’s “inner voice” or “visions”*) -* Smell |
| 40 | Embodied - Physical Sensation - Cold |
| 41 | Healing other |
| 42 | Direct - Communication with Others - Other “Beings” (Spirits/Entities/Guides) |
| 43 | Others - Physical sensations from other people  (e.g. someone’s knee is hurting and they feel it) |
| 44 | Senses - Direct Touch |

**References**

Aron, E. N., & Aron, A. (1997). Sensory-processing sensitivity and its relation to introversion and emotionality. *Journal of Personality and Social Psychology*, *73*(2), 345–368.

Capra, C., Kavanagh, D. J., Hides, L., & Scott, J. (2013). Brief screening for psychosis-like experiences. *Schizophrenia Research*, *149*(1–3), 104–107. https://doi.org/10.1016/j.schres.2013.05.020

Capra, C., Kavanagh, D. J., Hides, L., & Scott, J. G. (2015). Current CAPE-15: A measure of recent psychotic-like experiences and associated distress. *Early Interv Psychiatry*, *11*(5), 411–417. https://doi.org/10.1111/eip.12245

Gallagher, C., Kumar, V. K., & Pekala, R. J. (1994). The anomalous experiences inventory: Reliability and validity. *The Journal of Parapsychology*, *58*(4), 402.

Mikolajczak, M., Brasseur, S., & Fantini-Hauwel, C. (2014). Measuring intrapersonal and interpersonal EQ: The Short Profile of Emotional Competence (S-PEC). *Personality and Individual Differences*, *65*, 42–46. https://doi.org/10.1016/j.paid.2014.01.023

Rammstedt, B. (2007). The 10-item Big Five Inventory: Norm values and investigation of sociodemographic effects based on a German population representative sample. *European Journal of Psychological Assessment*, *23*(3), 193.

Wahbeh, H., Yount, G., Vieten, C., Radin, D., & Delorme, A. (2020). Measuring extraordinary experiences and beliefs: A validation and reliability study [version 3; peer review: 3 approved]. *F1000Research*, *8*(1741), 29. https://doi.org/10.12688/f1000research.20409.3

Waller, N. G., & Ross, C. A. (1997). The prevalence and biometric structure of pathological dissociation in the general population: Taxometric and behavior genetic findings. *Journal of Abnormal Psychology*, *106*(4), 499–510.

Waller, N., Putnam, F. W., & Carlson, E. B. (1996). Types of dissociation and dissociative types: A taxometric analysis of dissociative experiences. *Psychological Methods*, *1*(3), 300.

Zingrone, N. L., & Alvarado, C. S. (2001). The Dissociative Experiences Scale-II: Descriptive Statistics, Factor Analysis, and Frequency of Experiences. *Imagination, Cognition and Personality*, *21*(2), 145–157. https://doi.org/10.2190/K48D-XAW3-B2KC-UBB7
